# ﻿The identity of *Argyrialacteella* (Fabricius, 1794) (Lepidoptera, Pyraloidea, Crambinae), synonyms, and related species revealed by morphology and DNA capture in type specimens

**DOI:** 10.3897/zookeys.1146.96099

**Published:** 2023-02-07

**Authors:** Bernard Landry, Julia Bilat, James Hayden, M. Alma Solis, David C. Lees, Nadir Alvarez, Théo Léger, Jérémy Gauthier

**Affiliations:** 1 Muséum d’histoire naturelle de Genève, C.P. 6434, CH-1211, Geneva 6, Switzerland Muséum d’histoire naturelle de Genève Geneva Switzerland; 2 Florida Department of Agriculture and Consumer Services, Division of Plant Industry, Entomology Section, 1911 SW 34th Street, Gainesville, Florida, 32608, USA Florida Department of Agriculture and Consumer Services, Division of Plant Industry, Entomology Section Gainesville United States of America; 3 Systematic Entomology Laboratory, Beltsville Agriculture Research Center, Agricultural Research Service, U.S. Department of Agriculture, c/o National Museum Natural History, MRC 168, Smithsonian Institution, P.O. Box 37012, Washington, District of Columbia, 20013-7012, USA c/o National Museum Natural History Washington United States of America; 4 Natural History Museum, Cromwell Road, London, SW7 5BD, UK Natural History Museum London United Kingdom; 5 Département de Génétique et Evolution, Université de Genève, 30 quai Ernest Ansermet, 1205, Geneva, Switzerland Université de Genève Geneva Switzerland; 6 Muséum cantonal des sciences naturelles, Palais de Rumine, Place de la Riponne 6, 1005, Lausanne, Switzerland Muséum cantonal des sciences naturelle Lausanne Switzerland; 7 Museum für Naturkunde, Leibniz-Institut für Evolutions- und Biodiversitätsforshung, Invalidenstr. 43, 10115, Berlin, Germany Museum für Naturkunde Berlin Germany

**Keywords:** *Argyriacentrifugens* Dyar, *Argyriadiplomochalis* Dyar, *Argyriagonogramma* Dyar, COI barcodes, Crambidae, historical DNA, hybrid enrichment, species delimitation

## Abstract

In this study the aim was to resolve the taxonomy of several species of *Argyria* Hübner (Pyraloidea, Crambinae) with previously unrecognised morphological variation. By analysing the DNA barcode (COI-5P) in numerous specimens, the aim was to reconstruct phylogenetic relationships between species, to provide better evidence for synonymies, and to circumscribe their geographical distribution. Using an innovative DNA hybridisation capture protocol, the DNA barcode of the lectotype of *Argyrialacteella* (Fabricius, 1794) was partially recovered for comparison with the 229 DNA barcode sequences of *Argyria* specimens available in the Barcode of Life Datasystems, and this firmly establishes the identity of the species. The same protocol was used for the following type specimens: the *Argyriaabronalis* (Walker, 1859) holotype, thus confirming the synonymy of this name with *A.lacteella*, the holotype of *A.lusella* (Zeller, 1863), **syn. rev.**, the holotype of *A.multifacta* Dyar, 1914, **syn. nov.** newly synonymised with *A.lacteella*, and a specimen of *Argyriadiplomochalis* Dyar, 1913, collected in 1992. In addition, nine specimens of *A.lacteella*, *A.diplomochalis*, *A.centrifugens* Dyar, 1914 and *A.gonogramma* Dyar, 1915, from North to South America were sampled using classical COI amplification and Sanger sequencing. *Argyriagonogramma* Dyar, described from Bermuda, is the name to be applied to the more widespread North American species formerly identified as *A.lacteella*. Following morphological study of its holotype, *Argyriavestalis* Butler, 1878, **syn. nov.** is also synonymised with *A.lacteella*. The name *A.pusillalis* Hübner, 1818, is considered a nomen dubium associated with *A.gonogramma*. The adult morphology is diagnosed and illustrated, and distributions are plotted for *A.lacteella*, *A.diplomochalis*, *A.centrifugens*, and *A.gonogramma* based on slightly more than 800 specimens. For the first time, DNA barcode sequences are provided for the Antillean *A.diplomochalis*. This work provides a modified, improved protocol for the efficient hybrid capture enrichment of DNA barcodes from 18^th^ and 19^th^ century type specimens in order to solve taxonomic issues in Lepidoptera.

## ﻿Introduction

The name *Tinealacteella* Fabricius, 1794, and its synonyms, have been applied to small white moths of the genus *Argyria* Hübner collected in the New World since Fernald (1896) synonymised five species with it: *Argyriaalbana* (Fabricius), *Argyriapusillalis* Hübner, *Argyrialusella* (Zeller), *Argyriarufisignella* Zeller, and *Argyriapontiella* Zeller. Dyar (1903) then added *Argyriaabronalis* Walker as another synonym in a North American checklist. During the subsequent decades of the 20^th^ century, *A.rufisignella* and *A.pontiella* were removed from the list of synonyms of *A.lacteella* while *Argyriagonogramma* Dyar, 1914 was added to it as summarised by Munroe (1995). More recently, the name *A.lacteella* has been used, for example, in Moth Photographers Group (2022), BOLD (Ratnasingham and Hebert 2007), Scholtens and Solis (2015), and Landry et al. (2020). At the inception of this study, the BOLD database contained three widely separate lineages with specimens named *Argyrialacteella* and four with specimens identified as *Argyriacentrifugens* Dyar, 1914. Among the latter group, one lineage contained specimens collected in Florida, USA, and morphological examination of the holotype proved that their identification was erroneous.

Thus, because morphological and DNA barcode variation was observed in *Argyria* specimens that otherwise share a similar (ca. 11 mm) wingspan and previously unrecognised external diagnostic characters, we found it necessary to try to fix the identity of *A.lacteella* and the species similar to it, and to better understand their synonymy and geographical distribution. We aimed to do that by integrating both the COI barcode data available in BOLD and the type specimens of the species as well as those pertaining to synonymised names.

Until recently it has been impossible to recover genetic information from old museum specimens because the DNA they contain is degraded and occurs in very low quantities compared to contaminant DNA from other organisms (Card et al. 2021). It has sometimes been possible to recover short DNA barcodes at the cost of laborious multiple PCRs (Hernández-Triana et al. 2014), but recent developments in both the ability to recover historic DNA, improved extractions and capture approaches, and the advent of high-throughput sequencing have opened the access to the genetic information of these specimens, allowing many new studies and the emergence of museomics (Raxworthy and Smith 2021). Among the various approaches developed to recover DNA from collection specimens, hybrid enrichment methods seem to be the most efficient (Raxworthy and Smith 2021). These capture approaches can target different regions of the genome such as mitochondria (Zhang et al. 2020), exons (Bi et al. 2012), and conserved regions via conserved anchored hybrid enrichment (Espeland et al. 2018), ultraconserved elements (UCE) (Faircloth et al. 2012; Blaimer et al. 2016), and randomly distributed loci using the ddRAD approach (Suchan et al. 2016; Gauthier et al. 2020; Toussaint et al. 2021). These new methods make it possible to integrate old samples into modern genetic studies.

In this study, we adapted hybrid enrichment methods to target the COI barcode in old museum specimens. We designed probes along the entire nucleotide sequence of the COI barcode and synthesised our own RNA probes. We extracted historical DNA from four type specimens dating back to the 18^th^, 19^th^, and early 20^th^ centuries and an additional specimen collected in 1992. To successfully recover the COI barcode from this degraded, fragmented and contaminant-rich DNA, we combined hybrid enrichment capture and next-generation sequencing. We performed this sophisticated approach for these precious specimens because classic PCR amplification attempts were unsuccessful. In parallel, we amplified the DNA barcode for nine additional samples and integrated them with all available *Argyria* sequences in the BOLD database. Combining phylogenetic inferences, species delimitation approaches based on sequence data and morphology, we propose a new classification of several *Argyria* species. This study shows that innovative methods of museomics can solve complex taxonomic questions still debated. More generally, it reconciles the modernity of innovative molecular approaches with the biological heritage that museums have been preserving for centuries.

## ﻿Materials and methods

### ﻿Sources of information

The original description of *A.lacteella* and subsequent citation of the name by Fabricius (1794, 1798) were investigated, along with the original descriptions and subsequent citations of all other taxa/names treated here. The specimens examined came from the following institutions, in alphabetical order of acronyms:

**CMNH**Carnegie Museum of Natural History, Pittsburgh, USA;

**CUIC**Cornell University Insect Collection, Ithaca, New York, USA;

**FSCA**Florida State Collection of Arthropods, Gainesville, Florida, USA (curated with the MGCL);

**MFNB**Museum für Naturkunde, Berlin, Germany;

**MGCL** McGuire Center for Lepidoptera and Biodiversity, Gainesville, Florida, USA;

**MHNG**Muséum d’histoire naturelle, Geneva, Switzerland;

**NHMUK**Natural History Museum, London, UK;

**NMNH** (= USNM) National Museum of Natural History, Washington, D.C., USA;

**OUMNH**Oxford University Museum of Natural History, Oxford, UK;

**UCB**Essig Museum of Entomology, University of California, Berkeley, USA;

**VOB** V. O. Becker collection, Camacan, Bahia, Brazil;

**ZMUC**Zoological Museum of the University of Copenhagen, Denmark.

### ﻿Dissection

Specimens from which DNA was not extracted were dissected following Robinson (1976): abdomens were macerated in hot 10% aqueous KOH, cleaned, stained variously with Orange G, Chlorazol black, or eosin Y, and slide-mounted in Euparal.

### ﻿Illustrations

Photographs were taken with a variety of devices in five institutions (FSCA, MHNG, NMNH, NHMUK, ZMUC), including, at the MHNG (Figs 11–14, 20, 23–26), a Leica M205 binocular scope, a Leica DFC425 camera, and the Leica imaging software. The Visionary Digital imaging system was used at the NMNH. At the MHNG the photos were stacked using Zerene Stacker of Zerene Systems LLC and modified for better presentation using Adobe Photoshop Elements. At the FSCA, photographs were taken with a JVC digital camera KY-F75U 3-CCD with Leica Z16 Apo and Planapo 1.0× lenses, operated and stacked with Auto-Montage Pro v. 5.01.0005 (Syncroscopy, Synoptics, 2004). High-resolution genitalic photographs (Figs 17, 18, 27, 28) were taken at the MGCL with a Leica DM6B compound microscope with a Leica DMC6200 camera, and photographs were stacked and processed with Leica Application Suite X v. 3.7.0. Postprocessing was done with Adobe Photoshop Elements 11.

### ﻿Sampling

To ascertain the identity of *Argyrialacteella*, the DNA of its unique (as far as known) type specimen housed in the ZMUC was sampled from two legs. The DNA was sampled from the abdomen of the holotype of *Argyriaabronalis* (Walker, 1859), recorded as a synonym of *A.lacteella* (e.g., Munroe 1995) and deposited in the OUMNH. DNA was also sampled from one leg of the holotype of *A.lusella* (Zeller, 1863), which had been placed as a synonym of *A.lacteella* in the NHMUK, and from one leg and part of another for the holotype of *Argyriamultifacta* Dyar, 1914 deposited in the NMNH. In addition, the DNA of a specimen identified (by BL) as *Argyriadiplomochalis* Dyar, 1913 from the island of Anguilla, collected in 1992 and deposited in the CMNH, was also sampled in the same manner as the old holotypes just mentioned, and the DNA of two specimens of *A.diplomochalis* collected in 2021 on Saint Croix Island, US Virgin Islands, deposited in the MHNG, was sampled from one leg each using a Sanger protocol. The four specimens used here that were sequenced at the MFNB, but deposited in the MHNG, were sampled also from one leg each with a Sanger protocol. Additional specimens from the CMNH, CUIC, FSCA, MGCL, NMNH, UCB, and VOB were studied morphologically.

### ﻿DNA extraction and capture

In the MHNG, the DNA barcode sequence from specimen *Argyria “centrifugens*” DHJ02 (BOLD sample ID BIOUG27552-D08; JEH20210604A) (in reality, *Argyrialacteella*) captured at Gainesville (Florida, USA; deposited in FSCA) (29.6922°N, 82.3650°W) was used as reference for molecular work. Probes were designed using the 648 bp reference sequence via a sliding window of 108 pb with steps of 27 bp, providing an overlap of 83 bp. Using this approach, 21 probes were designed for the forward and 21 for the reverse direction. T7 promoters were added to each probe sequence. Final probe sets were ordered from Integrated DNA Technologies (IDT). The T7 reverse-complement sequence was annealed to the probe sets to allow transcription into RNA and biotinylation in a single reaction using HiScribe T7 High Yield RNA Synthesis Kit (New England Biolabs) followed by a Dnase treatment to avoid sample contamination by probe DNA during the capture, a purification using RNeasy kit (Qiagen) and Rnase inhibition using SUPERase-IN (Invitrogen). Concentrations of RNA probes were measured in a Qubit RNA HS assay (Thermo Fisher Scientific).

DNA extraction on historical samples were performed using PCR & DNA Cleanup Kit (Monarch). The protocol was adapted from Patzold et al. (2020) and aims to improve the recovery of small DNA fragments on the column with the addition of ethanol. In the non-destructive protocol, after a night in the Monarch gDNA Tissue lysis buffer with proteinase K (2 mg/ml final concentration), the abdomen of specimen CRA01 was treated with KOH, the genitalia were separated, and both genitalia and abdomen pelt were cleaned and mounted on slide following procedures mentioned in Landry and Becker (2021); the leg of specimen CRA02 was retrieved from the buffer and returned to the NHMUK where it is preserved in a vial underneath the specimen. In the destructive protocol the tissues were crushed (Table 1). The quality and concentration of purified DNA was assessed using a Qubit dsDNA HS assay (Thermo Fisher Scientific) and/or with Fragment Analyzer. Due to their low DNA concentration (Table 1), the samples were not diluted prior to the preparation of shotgun libraries, except for sample CRA01 (abdomen), which was diluted to ~ 27 ng/μL. A modified version of the protocol from Suchan et al. (2016) used in Toussaint et al. (2021) was applied for the preparation of shotgun libraries (detailed protocol in Suppl. material 1). Libraries were quantified using a Qubit dsDNA HS (Thermo Fisher Scientific) and pooled in equimolar quantities based upon their respective concentrations. For each probe set, forward and reverse, hybridisation capture for enrichment of shotgun libraries was performed following the protocol described in Toussaint et al. (2021). Sequencing was performed on Illumina Miseq Nano using a paired-end 150 protocol (Lausanne Genomic Technologies Facility, Switzerland).

**Table 1.** Data pertaining to specimens sampled with hyRAD and Sanger protocols at MHNG (CRA01-07), MFNB (BLDNA 065, 137, 138, 141), and MGCL (JEH20210604A-C).

ID	Sample	Year of collect	Extraction method	Tissue	DNA conc. (ng/ul)	#_reads	#_fragments	Mean fragment size (bp)	% COI barcode
**COI capture**									
CRA01	OUMNH Holotype *Argyriaabronalis* (Walker, 1859)	before 1859	non-destructive	abdomen	53.60	3.100,910	1.559,505	83.25	99.8
CRA02	NHMUK013696753 NHMUK Holotype *Argyrialusella* (Zeller, 1863)	before 1863	non-destructive	leg	0.40	8.889	1.583	81.88	96.3
CRA03	ZMUC Holotype *Argyrialacteella* (Fabricius, 1794)	1784–1789	destructive	leg	0.50	3.101	60	77.00	51.4
CRA04	CMNH*Argyriadiplomochalis* (Dyar, 1913)	1992	destructive	leg	2.94	862.323	183.175	103.51	100
CRA07	USNM*Argyriamultifacta* (Dyar, 1914)	1911	destructive	2 legs	0.17	14.538	10.686	49.40	93.1
**PCR amplification**									
CRA05	MHNG-ENTO-91928 MHNG*Argyriadiplomochalis* (Dyar, 1913)	2021	destructive	leg	0.81				100
CRA06	MHNG-ENTO-91929 MHNG*Argyriadiplomochalis* (Dyar, 1913)	2021	destructive	leg	0.79				100
BLDNA137	MHNG-ENTO-102922 *Argyrialacteella* (Fabricius, 1794)	2018	destructive	leg					100
BLDNA138	MHNG-ENTO-102923 *Argyrialacteella* (Fabricius, 1794)	2018	destructive	leg					100
BLDNA141	MHNG-ENTO-97427 *Argyriacentrifugens* (Dyar, 1914)	2018	destructive	leg					100
BLDNA065	MHNG-ENTO-85677 *Argyrialacteella* (Fabricius, 1794)	2004	destructive	leg					100
JEH20210604A	FSCA UF-FLMNH-MGCL 1112885 *Argyrialacteella* (Fabricius, 1794)	2021	destructive	leg					100
JEH20210604C	FSCA UF-FLMNH-MGCL 1112830 *Argyriagonogramma* (Dyar, 1915)	2020	destructive	leg					100
JEH20210604D	FSCA UF-FLMNH-MGCL 1112845 *Argyriagonogramma* (Dyar, 1915)	2015	destructive	leg					100

For additional samples, the DNA barcode was amplified by PCR. For CRA05 and CRA06 (Table 1), destructive DNA extraction was performed using QIAamp DNA Micro Kit (QIAGEN) and the DNA barcode was amplified by PCR using H02198 and COImod primers (Landry and Andriollo 2020) and sequenced using Sanger sequencing (Microsynth AG, Balgach, Switzerland). Samples BLDNA 65, 137, 138, and 141 (Table 1) were processed at the MfN: DNA was extracted using the Macherey-Nagel DNA extraction kit (Dürren, Germany), and molecular work followed the protocol described in Mey et al. (2021). Sequencing was done by Macrogen (The Netherlands) in both directions. Sequences were eye-checked and aligned using Phyde 0.9971 (Müller et al. 2005). The COI barcode region of samples JEH20210604A, ~C, and ~D was sequenced using standard barcoding primers and protocols (Hebert et al. 2004) by the FDACS-DPI Molecular Diagnostics Laboratory, in Florida, USA.

### ﻿COI locus reconstruction and phylogeny with existing data

Raw reads were cleaned using Cutadapt (Martin 2011) to remove barcodes, adapters and bases with a low quality, and quality was first checked using FastQC (Babraham Institute). Corresponding reads were first identified by BLASTn (Camacho et al. 2009) on the reference sequence and mapped using Geneious 6.0.3 Read Mapper (Kearse et al. 2012). Consensus sequences were generated keeping the most frequent bases and a minimum coverage of 3.

### ﻿Phylogenetic inferences

To investigate the phylogeny of *Argyria* species, the sequences from all the samples including the keyword “Argyria” were retrieved from the Barcode of Life Data System (**BOLD**) (Suppl. material 2). Newly generated and retrieved sequences were aligned using MAFFT (Katoh et al. 2002). The most likely nucleotide substitution model, i.e., GTR+G+I, has been identified using ModelFinder (Kalyaanamoorthy et al. 2017) implemented in IQ-TREE 2.0.5 (Minh et al. 2020). Phylogenetic inferences were performed in IQ-TREE 2.0.5 (Minh et al. 2020) and branch support were estimated using 1,000 ultrafast bootstraps along with 1,000 SH-aLRT tests (Guindon et al. 2010; Hoang et al. 2018). To avoid local optima, we performed 100 independent tree searches using IQ-TREE and selected the run showing the best likelihood score.

### ﻿Species delimitation and genetic distance

Three different methods were used to investigate species delimitation: Automatic Barcode Gap Discovery (ABGD) (Puillandre et al. 2012), Poisson Tree Processes (PTP) (Zhang et al. 2013) and General Mixed Yule-coalescent method (GMYC) (Pons et al. 2006). First, distance-based analysis ABGD was calculated using the K80 Kimura distance model and default parameters on the online platform (https://bioinfo.mnhn.fr/abi/public/abgd/abgdweb.html). Second, the single-locus species delimitation PTP method (Kapli et al. 2017) was used on the phylogeny excluding the outgroups *A.rufisignella* and *A.nummulalis*. Analyses were performed on the bPTP web server (https://species.h-its.org). The confidence of delimitation schemes was assessed using an MCMC chain of 10 million generations, a thinning of 100 and burn-in of 10%, and the partition with the best likelihood was kept. Third, the single threshold GMYC method was applied using the splits R package. The ultrametric tree required was generated using BEAST 1.10.4 (Suchard et al. 2018) with a GTR+G+I model as identified by ModelFinder (Kalyaanamoorthy et al. 2017), an uncorrelated relaxed clock with a lognormal distribution and an mtDNA COI substitution rate estimate of 0.0115 (Brower 1994). The species delimitation based on morphology has been compared to the delimitations based on molecular data. From the species described, the genetic p-distance was estimated between all pairs of samples using MEGA (Tamura et al. 2021) and summarised by species.

### ﻿Data availability

The sequence dataset is available on BOLD (DS-ARGYRIA). Raw reads are available on the NCBI SRA BioProject PRJNA914237.

## ﻿Results

### ﻿DNA recovery from historical samples

Historical DNA extraction showed different yields mainly related to the age of the specimens but also to the type of tissue and the extraction method, i.e., destructive or non-destructive (Table 1). Indeed, for the oldest sample, i.e., CRA03, captured between 1784–1789, it has been possible to extract DNA using a destructive approach on only one leg. For the two 19^th^ century samples, i.e., CRA01 and CRA02, a non-destructive approach was attempted. The sample with the lowest concentration of DNA is the CRA07 sample which was captured in 1911 and for which two legs were used destructively. Smaller fragments have been observed for the sample captured during the 18^th^ century, CRA03, and larger fragments for the sample CRA04 captured in 1992. Sample CRA07 is a special case since it was captured in 1911 but has small DNA fragments. For the more recent samples, captured after 2000, it was possible to extract enough DNA to perform a classical COI barcode amplification and Sanger sequencing (PCR amplification in Table 1). For the older samples, it has been necessary to develop a barcode capture approach because the amount of endogenous DNA was too low and initial trials at PCR amplification proved unsuccessful. This capture approach using probes designed along the COI barcode allowed NGS sequencing of 3.99 million reads in total with high heterogeneity between samples (Table 1). The number of reads seems correlated with the amount of DNA initially extracted. This heterogeneity in the amount of sequence recovered is then found throughout the bioinformatics analysis process until it impacts the percentage of barcode finally recovered. However, the capture approach was effective since it allowed the recovery of a sufficient proportion of the barcode to perform phylogenetic inferences for each of the samples, including the oldest sample, CRA03, and the particularly degraded CRA07 for which respectively more than 50% and 93.1% of the barcode was recovered.

### ﻿Phylogenetic inference and species delimitation

**Figure 1.**
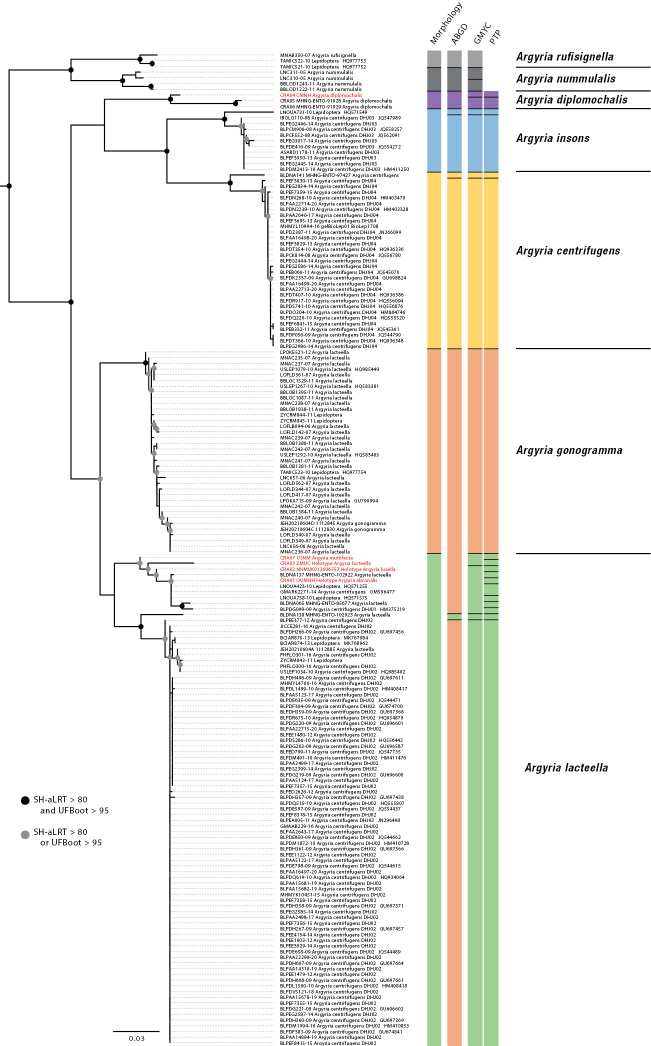
Phylogenetic inferences including 174 *Argyria* COI barcode sequences, i.e., 160 barcodes from BOLD, 9 COI sequences amplified by PCR (in bold), and 5 COI sequences obtained using capture from historical specimens (in red). Nodal support expressed in SH-aLRT and ultrafast bootstrap (UFBoot) is given as indicated in the caption except for nodes when SH-aLRT < 80 and/or UFBoot < 95. Species delimitation results including morphology, ABGD, GMYC, and PTP are indicated by different colours to represent the species proposed.

**Figure 2.**
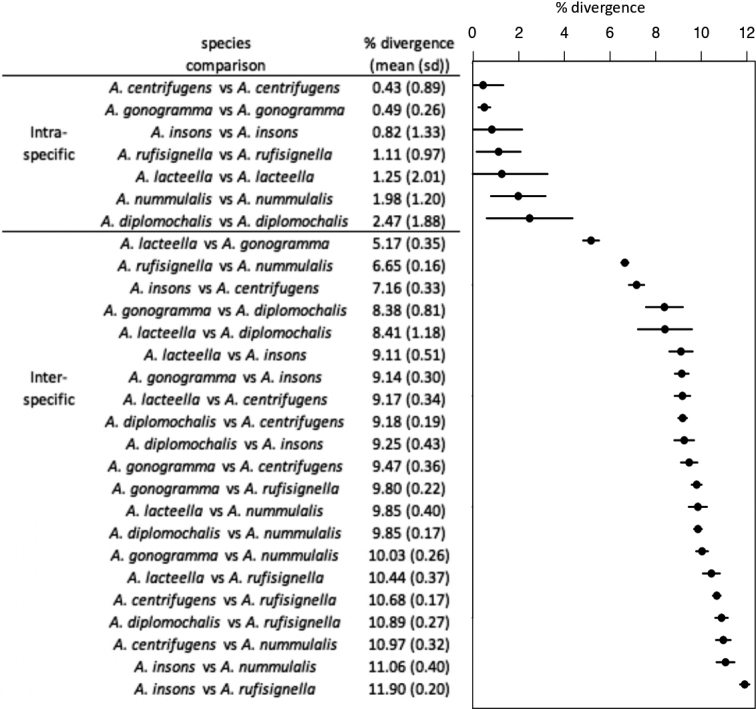
Mean genetic divergence (p-distance) between all samples from each species.

Phylogenetic inference has been performed on the whole COI barcode alignment including BOLD sequences, barcodes amplified for this study and sequences recovered using our historical DNA capture approach. The samples corresponding to the two species *A.rufisignella* and *A.nummulalis* have been used as outgroups, and their common node is well supported (Fig. 1). Then a well-supported node separates two clades, one includes the species *A.diplomochalis*, *A.insons* C. Felder, R. Felder & Rogenhofer, 1875, and *A.centrifugens* on one side and the species *A.gonogramma* and *A.lacteella* on the other (Fig. 1). Overall, the nodes separating the five species are also well supported. The three species delimitation analyses are consistent with each other and with morphology. The five species described and identified morphologically are almost all found by the species delimitation approaches, only the separation between *A.gonogramma* and *A.lacteella* has not been found in the ABGD approach based on the levels of divergence between sequences. However, the node separating the two species is well supported. Analysis of genetic divergence (p-distance) between each pair of individuals within and between species shows contrasting levels of divergence (Fig. 2). Within species, genetic divergence is low between 0.43% for *A.centrifugens* and 2.47% for *A.diplomochalis* for which we have only three samples. The distribution of genetic divergence then shows a gap with much higher values between samples belonging to different species and a percentage of divergence ranging from 5.17% to 11.90%. These results support the species identified using morphology and species delimitation approaches. Within each species the species delimitation approaches also identified additional separations mainly related to geographic divergences. The details of the divergences within species will be discussed next in the “Molecular diagnosis” section of each species.

### ﻿Taxonomic account

#### ﻿ Argyria lacteella

Taxon classificationAnimaliaLepidopteraCrambidae

(Fabricius, 1794)

073458B5-2643-5501-8879-94D08B834582

Figs 3 , 4 , 5 , 6 , 7 , 11 , 17 , 24 , 27 , 29 , 33

Tinea lacteella Fabricius, 1794: 313. Type locality: “Americaeinsulis” (USA Virgin Island of Saint Croix; see Remarks). Fernald 1896: 72, plate V figs 4, 6; Dyar 1903: 411; Grossbeck 1917: 126, probably referable to A.gonogramma, see Remarks; Schaus 1940: 400; Amsel 1956: 31, pl. 69 fig. 6, part of records, misspelled ‘lactella’; Munroe 1956: 127; Błeszyński and Collins 1962: 214; Zimsen 1964: 579, misspelled ‘lactella’; Kimball 1965: 234, part of records; Błeszyński 1967: 96; Jaume 1967: 2; De la Torre y Callejas 1967: 20; Alayo and Valdés 1982: 61; Tan 1984: 96 et seq., misidentification; Ferguson et al. 1991: 40, misidentification; Munroe 1995a: 35; Heppner 2003: 288, part of the records; Martinez and Brown 2007: 81, fig. 9, referable to A.gonogramma; Roque-Albelo and Landry 2010; Scholtens and Solis 2015: 54; Landry et al. 2020: 101, fig. 6B; Gibson et al. 2021: 29.
= albana (Fabricius, 1798 (Pyralis). Unnecessary replacement name.
= abronalis Walker, 1859: 969 (Zebronia??). Type locality: Brazil, Rio de Janeiro.
= lusella (Zeller, 1863: 51) (Catharylla). Type locality: St. Thomas Island [USA Virgin Islands]. Syn. rev.
= vestalis Butler, 1878: 494, 495. Type locality: Jamaica. Syn. nov.
= multifacta Dyar, 1914: 317. Type locality: Panama, Porto Bello. Syn. nov.

##### Type material examined.

**Figure 3.**
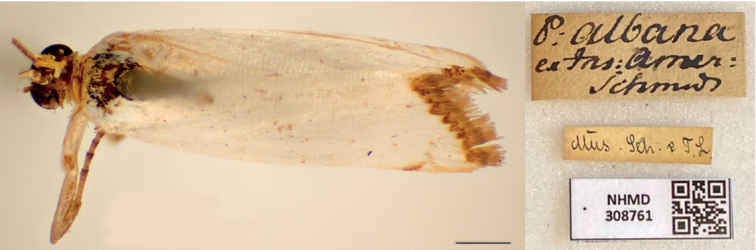
Lectotype of *Tinealacteella* Fabricius, 1794 (copyright of Natural History Museum of Denmark, ZMUC). Scale bar: 1 mm.

***Lectotype*** of *Tinealacteella* (Fig. 3), here designated, with label data as follows: 1- “P. albana | ex Ins: Amer: | ?Schmud?”, 2- “Mus[eum]. S[ehested] & T[oender] L[und], 3- “LECTOTYPE | *Tinealacteella* | Fabricius, 1794 | Des[ignated] by B. Landry, 2021”; deposited in ZMUC.

**Figure 4.**
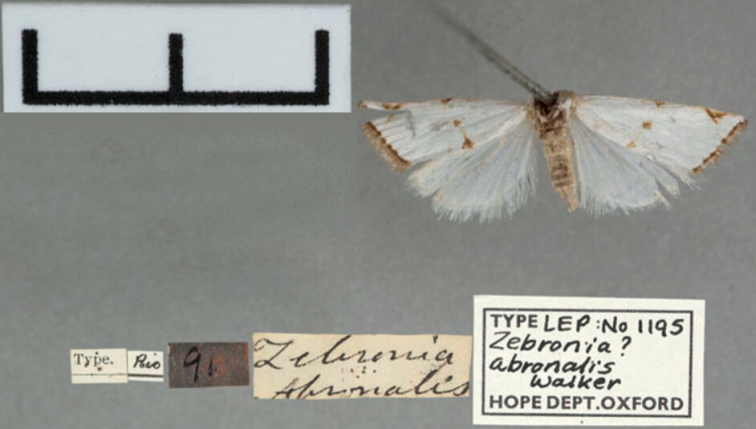
Holotype of *Argyrialacteella* synonym. Zebronia??abronalis Walker, 1859 (Oxford University Museum of Natural History; OUMNH). Scale bar: 10 mm.

***Holotype*** of *Zebronia*? *abronalis* (Figs 4, 24), with label data as follows: 1- “Type”, 2- “Rio”, 3- “91”, 4- “Zebronia | Abronalis”, 5- “TYPE LEP: No 1195 | Zebronia ? | abronalis | Walker | HOPE DEP[ARTMEN]T.OXFORD”; deposited in OUMNH.

**Figure 5.**
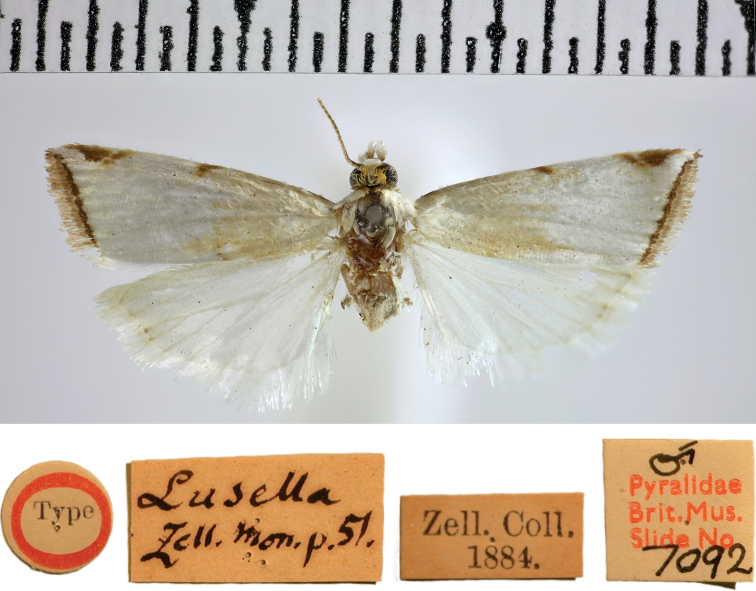
Holotype of *Argyrialacteella* synonym. *Catharyllalusella* Zeller, 1863 (NHMUK Trustees of the Natural History Museum).

***Holotype*** of *Catharyllalusella* (Fig. 5), with label data as follows: 1- “Type”, 2- Lusella | Zell[er]. Mon[ograph]. p.51.”, 3- Zell[er]. Coll[ection]. | 1884.”, 4- “♂ | Pyralidae | Brit.Mus. | Slide No. | 7092” | DNA voucher Lepidoptera B. Landry, n^o^ 00158 | NHMUK013696754 | MOLECULAR 215427977; deposited in the NHMUK.

**Figure 6.**
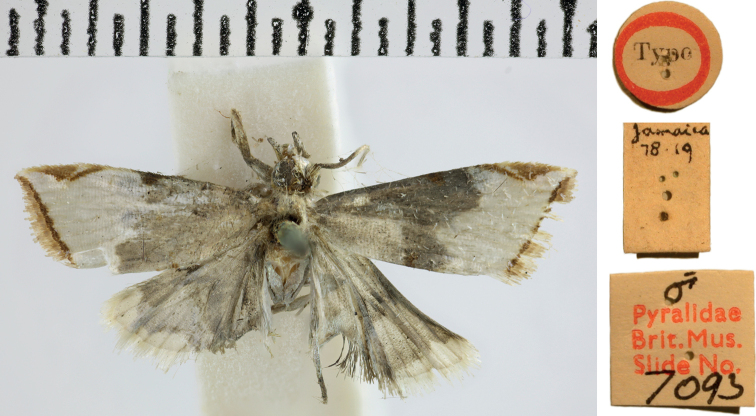
Holotype of *Argyrialacteella* synonym. *Argyriavestalis* Butler, 1878 (NHMUK Trustees of the Natural History Museum).

***Holotype*** of *Argyriavestalis* (Fig. 6), with label data as follows: 1- “Type”, 2- “Jamaica | 78. 19”, 3- ♂ | Pyralidae | Brit.Mus. | Slide No. | 7093 | NHMUK013696753”; deposited in the NHMUK.

**Figure 7.**
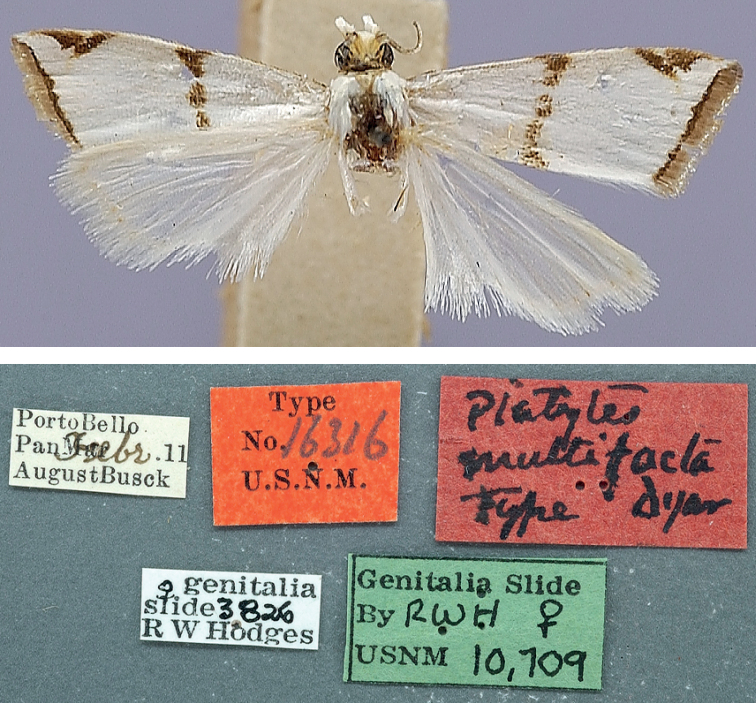
Holotype of *Argyrialacteella* synonym. *Argyriapusillalis* variety *multifacta* Dyar, 1914 (NMNH; wingspan: 12 mm).

***Holotype*** of *Argyriapusillalis* variety *multifacta* (Fig. 7) with label data as follows: 1- “PortoBello | Pan[ama]. Febr[uary]. [19]11 | AugustBusck”, 2- “Type | No.16316 | U.S.N.M.”, 3- “Platytes | multifacta | Type Dyar”, 4- “♀ genitalia | slide 3826 | R W Hodges”, 5- “Genitalia Slide | By RWH ♀ | USNM 10,709”; deposited in the NMNH. ***Paratypes*** of *Argyriapusillalis* variety *multifacta* with label data as follows: 1 ♂: 1- “PortoBello | Pan[ama]. Febr[uary]. [19]11 | AugustBusck”, 2- “♂ genitalia | slide, 29 Apr. ’32 | C.H. #29 | Genitalia slide | By ME ♂ | USNM 99,668”; 1 ♀: same data; 5 ♀♀, 2 ♂♂: same data except “Mar[ch]”; 2 ♀♀: 1- “RioTrinidad | Mar[ch]. [19]12 Pan[ama] | ABusck | coll”; 1 ♂: 1- “CorazolC[anal]Z[one] | Pan[ama] 3/24 [19]11 | AugBusck”, 2- “♂ genitalia | slide, 9 June. ’32 | C.H. #83” [slide not found]; deposited in the NMNH. [Note: the Tabernilla (Busck) and Corazol (Crafts) specimens were not found at NMNH]

##### Other specimens examined.

238 specimens (see Suppl. material 2).

##### Morphological diagnosis.

**Figure 8.**
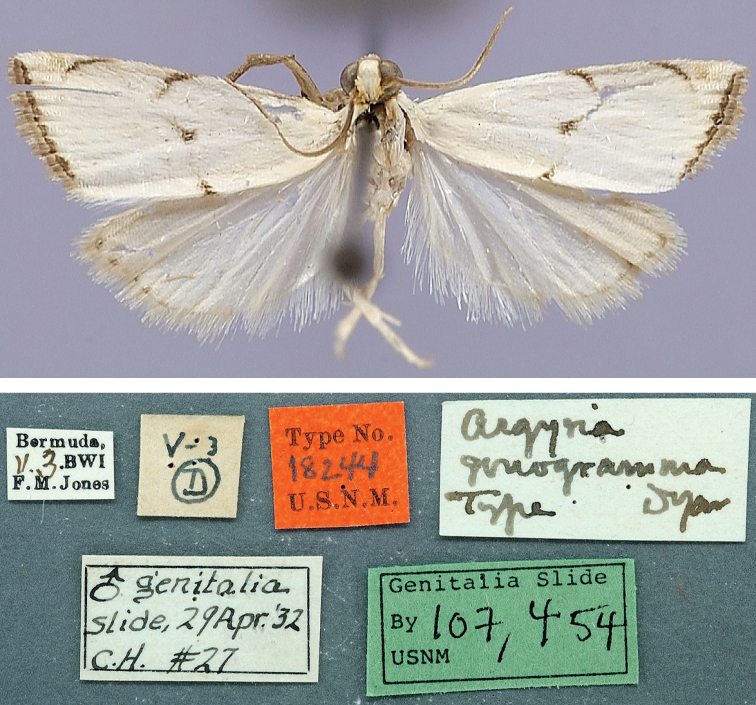
Habitus of *Argyria* type specimen (in NMNH) with labels underneath; holotype of *Argyriagonogramma* Dyar, 1915 (wingspan: 11 mm).

This is a small satiny white moth of 9.5–14 mm in wingspan. The forewing brown markings are median triangles on the costa and dorsal margin usually linked by a thin straight line sometimes slightly thicker on the discal cell as a spot, but sometimes inconspicuous, another triangle subapically on costa, usually separated by a thin white line from a short oblique dash anteriorly, and a wavy terminal line (Figs 3–6, 11). There are also specimens of *A.lacteella* with a complete median fascia (Fig. 7) in the South of the distribution of the species, in Panama (holotype of synonym *A.multifacta*), French Guiana, Bolivia, and Brazil. In forewing markings *A.lacteella* differs from *A.gonogramma* (Figs 8, 12), which usually has a well-marked darker, blackish-brown spot on the discal cell, linked by a thin, curved line to a short diagonal bar on costa and a thin triangle on the dorsal margin, and without a clear costal triangle subapically. In forewing markings *A.lacteella* is most similar to *A.centrifugens* (Fig. 10), which is generally bigger (14–19 mm in wingspan) and which has the line anteriad to the subapical costal triangle curved to reach the costa at right angle whereas that line in *A.lacteella* runs obliquely into the costa. In male genitalia (Fig. 17) this species differs from the most similar *A.gonogramma* by the basal projection of the valva that is slightly longer and bent mesad at right angle whereas it is just barely curved in *A.gonogramma* (Figs 16, 18). The cornuti on the vesica also are smaller and thinner in *A.lacteella* compared to those of *A.gonogramma*. In female genitalia *A.lacteella* (Figs 24, 27) is also most similar to *A.gonogramma* (Fig. 28), but *A.lacteella* has two “pockets” anterolateral of the ostium bursae, whereas *A.gonogramma* has one continuous pocket anterior of the ostium.

##### Molecular results.

Phylogenetic inference based on COI barcode alignment reveals a large clade grouping the *A.lacteella* samples. This clade is relatively homogeneous since the percentage of divergence within this species remains low with an average of 1.25% (Fig. 2). It is then divided into two clades also identified by the species delimitation approaches. The first one is mainly composed of samples from South America, i.e., Brazil, French Guiana, Argentina, Colombia, but also from the Galapagos Islands. The different intraspecific delimitations identified by the species delimitation approaches within this clade are therefore certainly related to geographical divergence. The historical samples originating from Panama and the United States Virgin Islands belong to this clade as well. The barcodes of these samples are not complete (Table 1), the missing may induce phylogenetic artefacts due to long-branch attraction. A molecular analysis focused on this species including more localities but especially more loci could clarify this situation. The second cluster is composed of a clade of samples from the US on one side and a very large clade of samples from Costa Rica on the other. The latter shows a very low level of variation.

##### Distribution.

Widespread in the Western Hemisphere from the US State of Florida north to Alachua County in the north, across Central America and the Antilles, in South America to Argentina in the south, as well as on the Galápagos Islands (Fig. 33).

##### Remarks.

Fabricius (1798) changed the name of his *lacteella* (1794) with another (*albana*). The reason for this is unrecorded and remains unclear, but this is possibly because Fabricius (1798: 476, spelling it “*lactella*”) incorrectly considered *lacteella* to be a homonym of *Tinealactella* Denis & Schiffermüller, 1775 (a synonym of *Endrosissarcitrella* (Linnaeus)). Also, he may have corrected ‘improper’ names as in his treatments of *Tineacompositella* Fabricius, 1794 and *T.tapetzella* Linnaeus, 1758, both without putative ‘homonyms’ and respectively renamed *Pyraliscomposana* and *P.tapezana* (Fabricius, 1798: 480), or he felt that the exact orthography of any name was not so important.

The first label associated with the lectotype of *A.lacteella* (Fig. 3) reads “P[yralis]. albana | ex Ins. Amer: | Schmidt”. The second line of this label means “from the American Islands” while the name of the third line refers to the collector of the specimen, who, according to Zimsen (1964) was either Adam Levin Smidt, a custom-house officer, or Johan Christian Schmidt, a surgeon. Both lived on the island of St Croix, which was at that time a Danish possession (T. Pape, pers. comm. to BL, 18 August 2022). The second label associated with this type specimen refers to the collection of Ove Ramel Sehested and Niels Tønder Lund who lived in Copenhagen and were pupils and friends of Fabricius (Baixeras and Karsholt 2011).

The locality of origin is an additional complication associated with *A.lacteella*. The locality of Fabricius’ *Pyralisalbana* (1798) is mentioned as “*Americaeinsulis*” [American islands] whereas that of *A.lacteella* (1794) is “*Americaemeridionalisarboretis*” [South American arboretum]. Given that “Dr. Pflug” is mentioned in the original description of *A.lacteella*, it is reasonable to conclude that “*Americaeinsulis*” was a correction for “*Americaemeridionalisarboretis*”. This is because Paul Gottfrid Pflug (1741–1789), a medical doctor, lived in the Caribbean island of Saint Croix (United States Virgin Islands) during the last five years of his life, where he collected insects that he sent to Denmark. He is mentioned often by Fabricius as a specimen collector (O. Karsholt, pers. comm. to BL, 3 June 2021). Therefore, *A.lacteella*/*albana* is from an American island (*Americaeinsulis*) that is probably Saint Croix.

As confirmed by Copenhagen Museum former curator Ole Karsholt and present curator Thomas Pape, only one type specimen presently exists for *lacteella*/*albana* (Fig. 1) and because *albana* is best considered as an unjustified replacement name, the type of *Pyralisalbana* Fabricius, 1798 is the same as that of *Tinealacteella* Fabricius, 1794. This specimen is without an abdomen and is designated as the lectotype upon the recommendation of curator T. Pape, who wrote (pers. comm. to BL, 7 June 2021): “As Fabricius does not indicate the number of specimens, I would consider a lectotype designation as appropriate, unless this has already been done by referring to this specimen as “the type” or something similar.” Such a designation also serves to stabilise the identity of the species name laden with confusion caused by Fabricius himself.

The type specimen of *A.lacteella* (Fig. 3) is badly rubbed, lacking most scales on the head, and some on the thorax and forewings as shown by the denuded anal vein on the right forewing. It also lacks most of the diagnostic brown markings of the forewing, notably the subapical triangle on the costa, but the terminal zigzagging brown line is almost complete and there are a few brown scales in the position of the median spot and fewer brown scales still on the dorsal margin medially.

Munroe (1995: 35) stated that *Argyriaabronalis* is a nomen dubium, but this is incorrect as a female type (Figs 4, 24) is in the Oxford University Museum of Natural History (also figured at https://www.oumnh.ox.ac.uk/collections-online#/item/oum-catalogue-3393). The forewing markings, female genitalia morphology and DNA barcode all concur to validate the synonymy of *A.abronalis* with *A.lacteella*. The species was described from the female sex, without indication or indirect evidence of more than one specimen; therefore, the OUMNH specimen is considered the holotype.

The original description of *Catharyllalusella*Zeller (1863) explicitly mentioned one female only, described from the island of Saint Thomas, US Virgin Islands. Thus, the male sign on this holotype’s slide number label is incorrect (Fig. 5). The specimen was dissected and although the genitalia dissection was not thoroughly cleaned, the visible morphological characters agree with those of *A.lacteella*. The forewing markings lack the median triangle of the dorsal margin and any indication of a median transverse line, as in the holotype of *A.lacteella*, but the subapical triangle on the costa and especially the COI barcode obtained clearly show that *C.lusella* syn. rev., should be considered a synonym of *A.lacteella*. The name had been synonymised by Fernald (1896), considered a synonym also by Schaus (1940), but considered valid again by Błeszyński and Collins (1962) and Munroe (1995; misspelled “*lusalla*”).

The original description of *Argyriavestalis* does not mention more than one specimen and the NHMUK does not hold additional specimens with these label data; therefore, this specimen is considered the unique holotype. It is a lightly marked, damaged, dissected male (Fig. 6); the dissection clearly shows the curved projection at the base of the valva that is diagnostic for *A.lacteella*; therefore, the name *A.vestalis* syn. nov. is considered a synonym of *A.lacteella*.

*Argyriamultifacta* was described as a variety of *A.pusillalis* for which “All the specimens have the median band continuous across the wing” (Dyar 1914: 317). Among a series of specimens mentioned from several localities in the Panama Canal zone, one is recorded as Type with the type number and label data mentioned above (Fig. 7). Although this holotype shows a conspicuous and almost continuous median band on the forewing, thus revealing strong variation in that respect in the species, other wing characters, size, and the COI barcode data point to the synonymy of *A.multifacta* syn. nov. with *A.lacteella*.

This species evidently became established in Florida, USA in the 1970s and consequently, earlier records from Florida (Grossbeck 1917; Kimball 1965) are believed to be wrong and referable to *A.gonogramma*. The earliest specimen known to us was collected in Miami-Dade County, Fuchs Hammock near Homestead, by T.S. Dickel on 31 August 1979 (MGCL catalogue no. 1112898, slide 6219, deposited in FSCA). The species rapidly spread across the state, as shown by first collection years in other vouchered counties: 1983: Highlands, Monroe, Orange; 1986: Collier, Manatee; 1987: Volusia; 1988: Lee; 1990: Pinellas; 1991: Hernando; 2000: Brevard; 2003: Marion; 2005: Alachua; 2012: Indian River; 2013: Levy (FSCA, MGCL). The collection of *A.gonogramma* in Florida decades before *A.lacteella* strongly suggests that the latter species is non-native and that it invaded in the given time frame (see Remarks for *A.gonogramma*).

Amsel (1956: 31, pl. 69 fig. 6) mentions the species from specimens sporting a wingspan of 12–18 mm, and although his illustration probably represents *A.lacteella*, no specimens examined of that species were found to reach a wingspan of more than 14 mm.

The vesica of a male specimen from Florida, USA (not illustrated here) was successfully everted by J. Baixeras, who wrote the following to BL on 17 October 2022: “After a lot of manipulation I was able to evert what seems like a rather tubular vesica bearing a single row of non-deciduous cornuti tightly arranged like in a “gun charger” mode. The vesica seems to be somewhat convoluted at the base (I do not think it an artefact), then straight. The cornuti are extended all over the length of the vesica except in the terminal part, close to the genital opening. The basal convolution is interesting and, if my surmise is correct, should be correlated with some structure in the female, either a pocket, broadening sclerotisation or, in some cases, some corrugated area allowing expansion during insertion.” The basal convoluted bend at the base of the vesica reflects the shape of the basal section of the female ductus bursae, which is indeed corrugated (Figs 24, 27).

#### ﻿ Argyria gonogramma

Taxon classificationAnimaliaLepidopteraCrambidae

Dyar, 1915

AA15006B-7D5D-5AA2-8CC0-4042E95F1283

Figs 8 , 12 , 13 , 15 , 16 , 18 , 28 , 30 , 34

Argyria gonogramma Dyar, 1915: 87–88. Type locality: Bermuda. Błeszyński and Collins (1962: 213).
= pusillalis Hübner, 1818: 30, [36], [38], figs 167, 168. Type locality: [USA, Maryland] Baltimore. Nomen dubium.
= *pussillalis* [sic] Hübner, 1818: 28; original misspelling.
Argyria lacteella (Fabricius, 1794): Fernald 1896: 72, plate V fig. 5; Grossbeck 1917: 126; Kimball 1965: 233; Tan 1984: 96 et seq.; Ferguson et al. 1991: 40; Munroe 1995: 35 (in part); Martinez and Brown 2007: 81, fig. 9.

##### Type material examined.

***Holotype*** ♂ (Figs 8, 15, 16), with label data as follows: 1- “Bermuda, | 11.3.BWI | F.M. Jones”, 2- “V-3 | D”, 3- “Type No. | 18244 | U.S.N.M.”, 4- “Argyria | gonogramma | Type Dyar”, 5- “♂ genitalia | slide, 29Apr[il].’32 | C.H. #27”, 6- “Genitalia Slide | By 107,454 | USNM”; deposited in the NMNH.

##### Other specimens examined.

411 specimens (see Suppl. material 2).

##### Morphological diagnosis.

**Figure 9.**
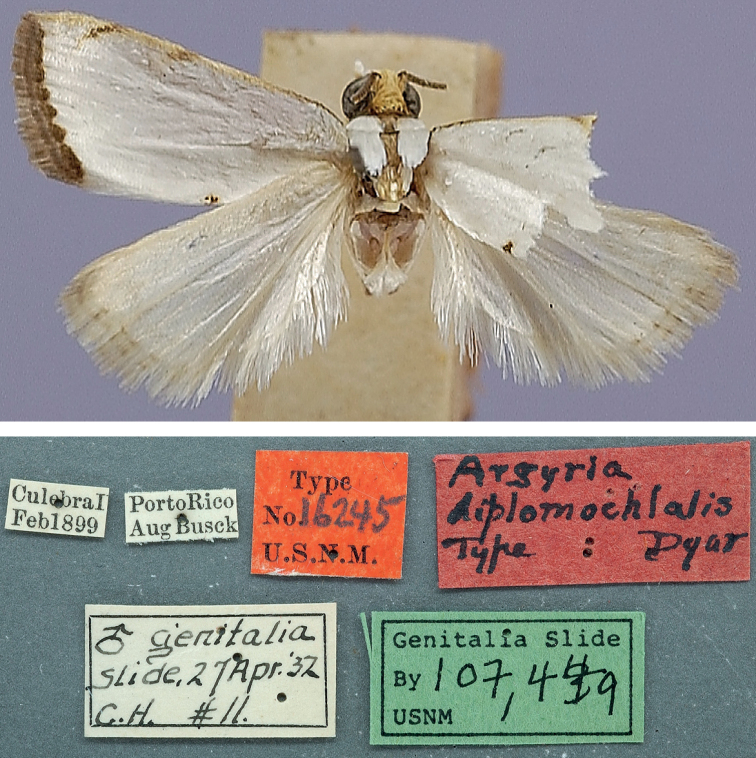
Habitus of *Argyria* type specimen (in NMNH) with labels underneath; lectotype of *Argyriadiplomochalis* Dyar, 1913 (wingspan: 11 mm).

In this small satiny-white moth measuring between 10.5 and 13.5 mm in wingspan, the median markings of the forewing (Figs 8, 12) usually include a well-marked blackish-brown spot on the discal cell that is connected by curved lines to an oblique bar on the costa and a thin triangle on the dorsal margin. On the forewing costa, subapically, a thin curving bar is not followed by a triangle, but usually by 1–3 horizontal lines reaching the terminal margin below the apex. Relatively dark brown forms, with less contrasting markings (Fig. 13) have been collected in Alabama, Florida, and Louisiana (CUIC, FSCA, MHNG, NMNH) from December to April. In forewing markings this species is closest to *A.lacteella* (Fig. 11) in which there usually is a clear subapical triangle on the costa and for which the median spot, if present, is paler brown and usually smaller than the costal and dorsal triangles. In the absence of a subapical triangle on the forewing costa *A.gonogramma* is also similar to *A.diplomochalis* (Figs 9, 14), which, however, doesn’t have any indication of a median spot or of any line between the median spot of the dorsal margin and the costa. In the male genitalia *Argyriagonogramma* (Figs 15, 16, 18) has the basal projection of the valva shorter than that of *A.lacteella* (Fig. 17) and just barely curving (Fig. 16); the cornuti on the vesica are also longer and thicker than those of *A.lacteella*. In the female genitalia (Fig. 28) only one wide, sclerotised pocket can be found anterior of the ostium bursae, whereas *A.lacteella* (Figs 24, 27) has two pockets in the same area.

**Figure 10.**
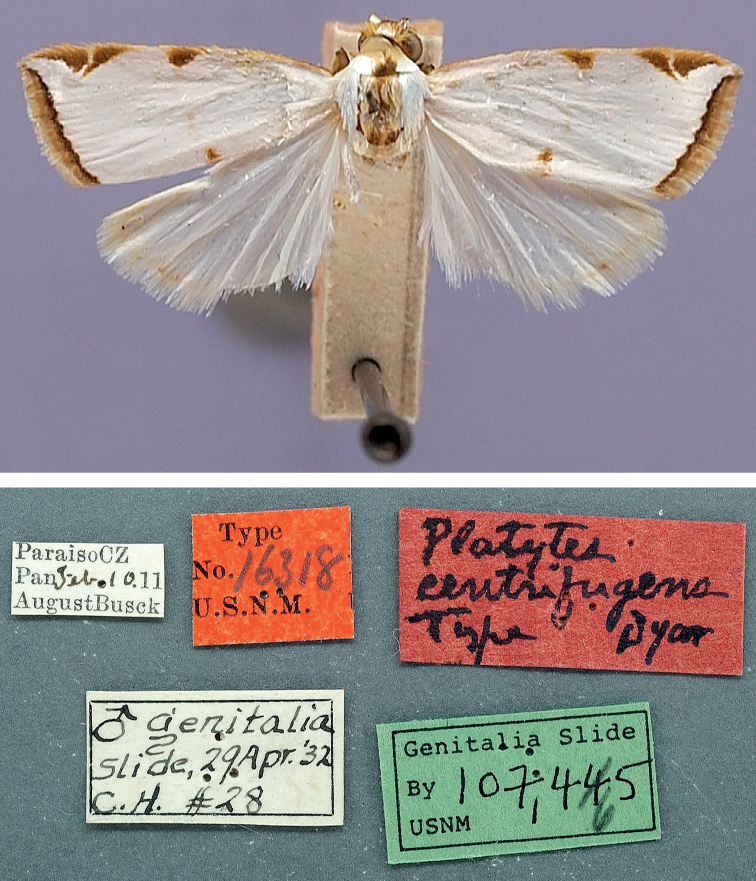
Habitus of *Argyria* type specimen (in NMNH) with labels underneath; holotype of *Argyriacentrifugens* Dyar, 1914 (wingspan: 16 mm).

**Figure 11.**
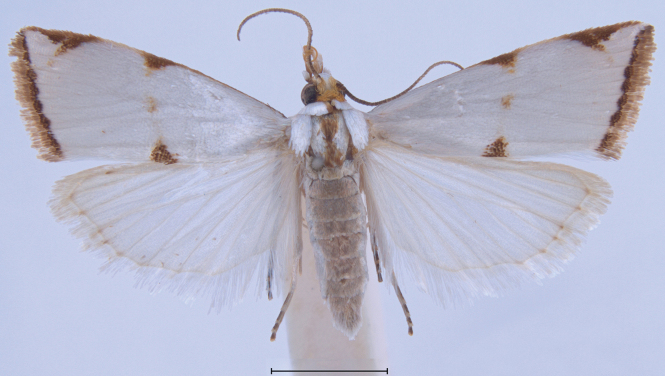
Habitus of additional *Argyria* specimen. *Argyrialacteella* (Florida, Putnam Co., FSCA). Scale bar: 2.5 mm.

**Figure 12.**
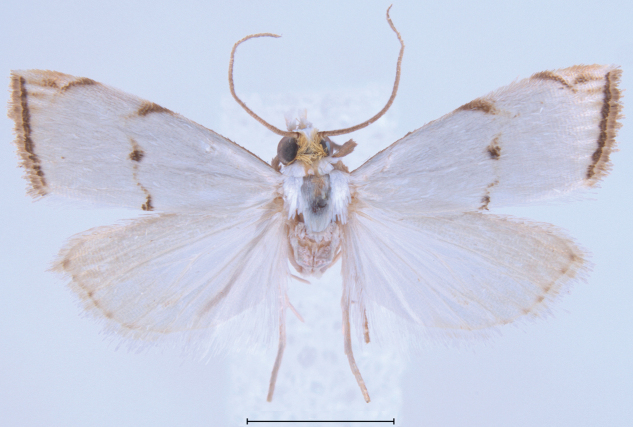
Habitus of additional *Argyria* specimen. *Argyriagonogramma* (Florida, Seminole Co., MHNG). Scale bar: 2.5 mm.

**Figure 13.**
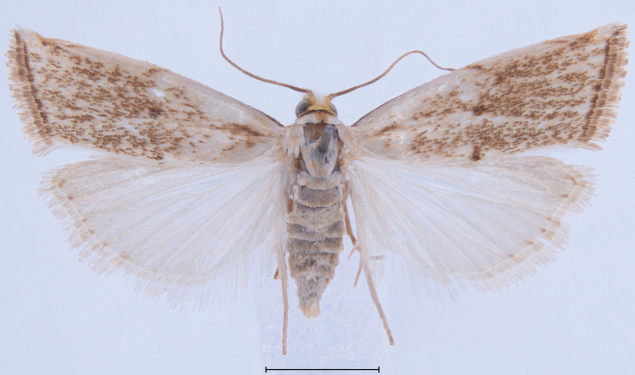
Habitus of additional *Argyria* specimen. *Argyriagonogramma* (dark form, Louisiana, Calcasieu Co., MHNG). Scale bar: 2.5 mm.

**Figure 14.**
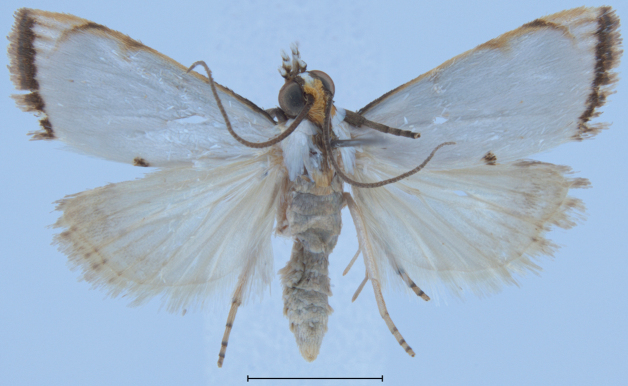
Habitus of additional *Argyria* specimen. *Argyriadiplomochalis* (St Croix Island, MHNG). Scale bar: 2.5 mm.

**Figure 15.**
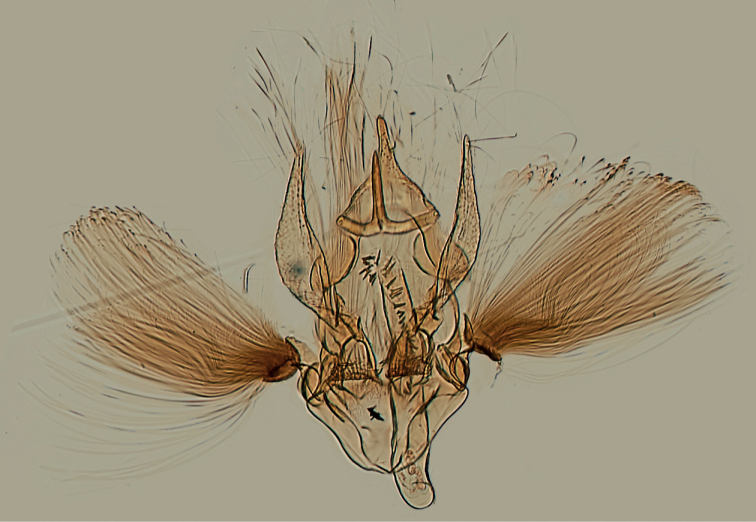
Male genitalia of *Argyriagonogramma* holotype (NMNH). Whole genitalia.

**Figure 16.**
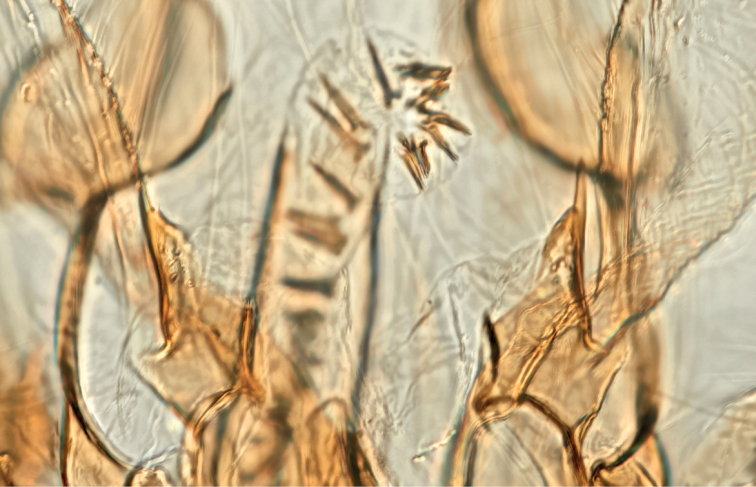
Male genitalia of *Argyriagonogramma* holotype (NMNH). Close-up of bases of valvae with tip of phallus in middle.

**Figure 17.**
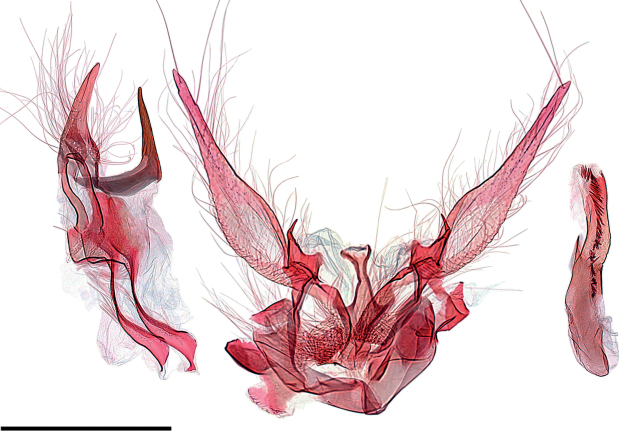
Male genitalia of *Argyria* species. *Argyrialacteella* (Florida, Alachua Co.). In FSCA. Scale bar: 500 µm.

**Figure 18.**
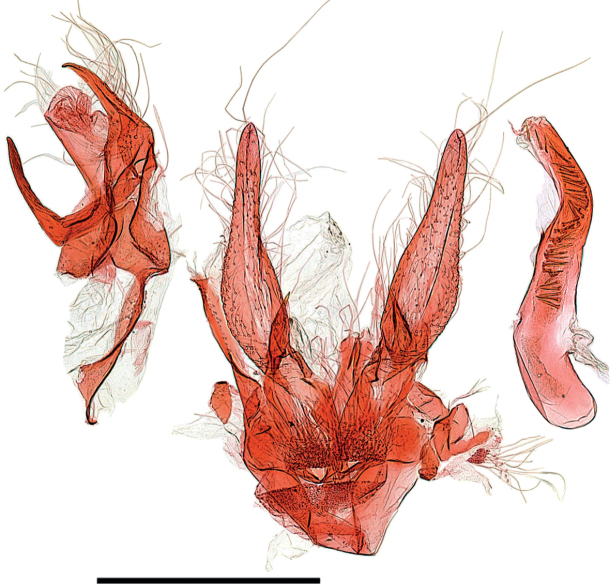
Male genitalia of *Argyria* species. *A.gonogramma* (Florida, Wakulla Co.). In FSCA. Scale bar: 500 µm.

##### Molecular results.

Phylogenetic inference reveals that *Argyriagonogramma* constitutes a homogeneous clade. The monophyletic clade is identified in both GMYC and PTP species delimitation approaches, but it is not found in the ABGD approach and is grouped with *A.lacteella* (Fig. 1). This clade shows very low genetic variability within the COI barcode with an average intraspecific divergence of only 0.49% (Fig. 2). This low genetic diversity may be the result of different evolutionary processes, including recent colonisation. This species is mainly present in the US where it overlaps with *A.lacteella* in Florida (Figs 33, 34).

##### Distribution.

Bermuda, Bahamas, widespread in the Eastern USA, from North Carolina in the North to the south of Florida, west to eastern Texas (Fig. 34).

##### Remarks.

The specimen of *A.gonogramma* labelled ‘Type’ in the NMNH is considered the unique holotype; the species’ description (Dyar 1915) doesn’t indicate multiple specimens.

*Argyriapusillalis* Hübner is associated here with *A.gonogramma* and not with *A.lacteella* as in Munroe (1995) because at the latitude of Baltimore, Maryland, U.S.A., the type locality of *A.pusillalis*, only the superficially similar *A.gonogramma* or *A.rufisignella* (Zeller, 1872) could occur. *Argyrianummulalis* Hübner, 1818 is also known to occur in the eastern USA at the latitude of Baltimore, but this species lacks any median markings across the forewing, unlike the illustration of *A.pusillalis*. The name *A.pusillalis* is considered a *nomen dubium* because the original description and illustration associated with it do not allow a conclusive determination. Hübner’s collection was deposited in the Naturhistorisches Museum Wien, Vienna, Austria, which was destroyed by fire in 1835 (Horn et al. 1990). However, although type specimens of some of Hübner’s Noctuidae species have recently been discovered in this museum (Gabor and László Ronkay, pers. comm. to BL, 11 April 2022), a search for a type specimen of *A.pusillalis* was not successful (S. Gaal, pers. comm. to BL, 10 August 2022). This issue could be settled by the designation of a neotype, but we refrain from doing that in order to avoid more instability in the nomenclature of this group. Also, we believe that it should be done in conjunction with a taxonomic revision of *A.rufisignella*, at the least.

*Argyriapusillalis* was originally named “*pussillalis*” (Hübner 1818: 28), then mentioned as “*pusillalis*” on page 30 and on two indices (pages [36] and [38]), and finally as “*pussillalis*” again on the plate with the illustrations. Given that “*pusillus*” is Latin for small, it seems reasonable to believe that the original spelling “*pussillalis*” was in error.

*Argyriagonogramma* is a North American native species that was previously misidentified as *A.lacteella* and that has been collected in the United States since the late 1800’s. The earliest specimens in the NMNH were collected by C.V. Riley from Ar[t]elier, FL, 1882 and N.[orth]C.[arolina] (undated). Another specimen collected by Boll in Texas (collection date unknown) was identified by “Rag[onot] \[18]86”, and then by “CVR[iley]at the B. Mus. \[18]87”.

That this species is native to the Southeastern U.S., or at least was established long before *A.lacteella*, is shown by earlier collecting dates for specimens in the FSCA and MGCL. For example: Florida, Sarasota Co.: 1951, Alachua Co.: 1960, Volusia Co.: 1962, Okaloosa Co.: 1963, Texas: 1978, Louisiana: 1979.

The earliest record of *A.lacteella* in 1979 in the USA (Florida) supports the conclusion that Tan (1984), although referring to *A.lacteella*, was in fact dealing with *A.gonogramma*. Tan’s (1984) unpublished MSc thesis provided a description of larvae, with setal maps, which were reared from egg to adult on St. Augustine grass (*Stenotaphrumsecundatum* (Walt.) Kuntze; Poaceae). Tan (1984) further mentions that the early instar larvae eat the upper epidermis only and when not feeding, larvae hide in shelters made of leaves attached with silk, wherein moulting occurs. Tan (1984) also records the construction by the mature larva of a “small, compact silken case covered with frass and tiny pieces of chewed grass for pupation.”

Based on collected series of specimens both *Argyriagonogramma* and *A.lacteella* now occur in sympatry and fly on the same dates in Florida, for example at Archbold Biological Station in Highlands County or in Pinellas County.

Fernald (1896) treated this species under *A.lacteella* (pl. V fig. 5), whereas his other illustrations on the same plate (Figs 4, 6) represent the true *A.lacteella*. Grossbeck (1917), Kimball (most or all records, 1965), and Martinez and Brown (2007) all treated this species under *A.lacteella*. Melanic specimens collected in winter months account for the specimens of “*A.diplomochalis*” cited by Kimball (1965). This colouration variant may represent an adaptation for hiding in dry grass during the winter months and/or to obtain extra calories from the sun to allow biological activity.

The single moth at the basis of the Vermont record has been dissected and is correctly determined, but it is far outside the range since we know of no other record of *A.gonogramma* north of North Carolina. It was collected in sandplain habitat (M. Sabourin, pers. comm. to JH, 29 August 2022), which is consistent with the species’ habitat preference in Florida.

#### ﻿ Argyria diplomochalis

Taxon classificationAnimaliaLepidopteraCrambidae

Dyar, 1913

256937A6-D27D-550C-9464-135CD76242F0

**Figure 20.**
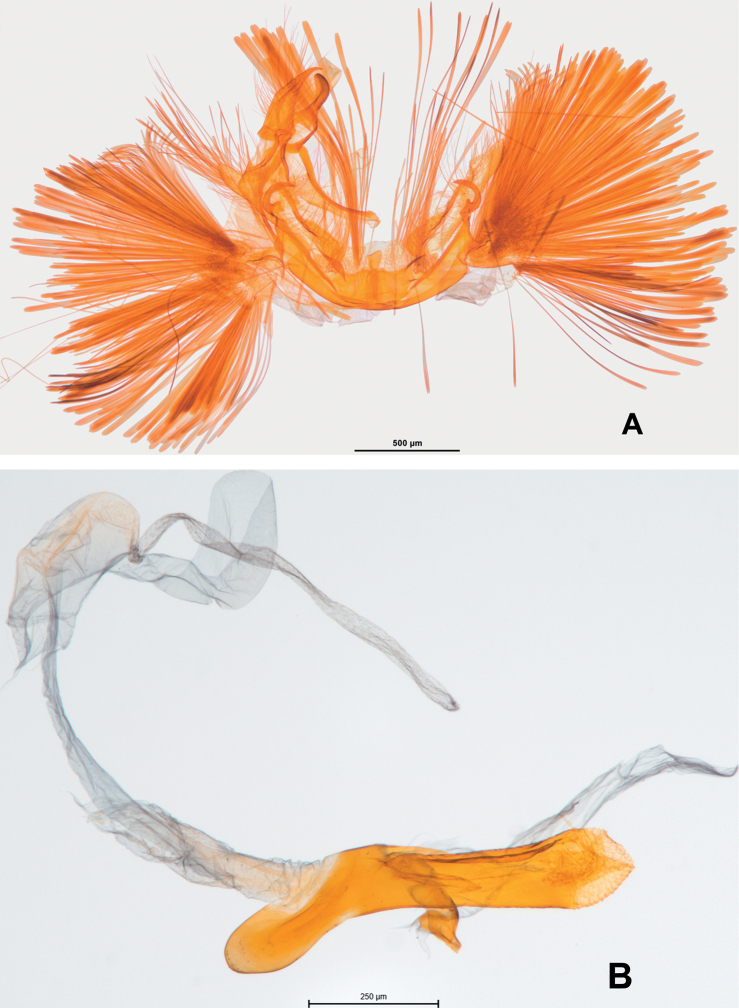
Male genitalia of *Argyriadiplomochalis*. Specimen from St Croix Island (MHNG-ENTO-91929) (**A**) with phallus detached (**B**).

Figs 9 , 14 , 19 , 20 , 25 , 31 , 35

Argyria diplomochalis Dyar, 1913: 113. Type locality: [USA] Culebra Island, Puerto Rico. Błeszyński and Collins 1962: 213; Kimball 1965: 234, US specimens misidentified; Błeszyński 1967: 96; De la Torre y Callejas 1967: 20; Alayo and Valdés 1982: 61; Munroe 1995: 35.
Argyria diplamachalis [sic]: Schaus, 1940: 400.

##### Type material examined.

***Lectotype*** ♂ (Fig. 9), here designated, with label data as follows: 1- “CulebraI[sland] | Feb[ruary]1899”, 2- “PortoRico | Aug[ust.] Busck”, 3- “Type | No.16245” | U.S.N.M.”, 4- “Argyria | diplomochalis | Type Dyar”, 5- “♂ genitalia | slide, 27 Apr[il].[19]’32 | C.H. #11.”, 6- “Genitalia Slide | By CH | 107,449 | USNM”, 7- “Lectotype | Argyria | diplomochalis Dyar, 1913 | Des[ignated] by M.A. Solis, 2022”, deposited in the NMNH. ***Paralectotypes*** (8 ♂, 1 ♀), here designated with label data as follows: 3 ♂♂: 1-“Culebra I[sland] | Feb[bruary]1899”, 2- “Porto Rico | Aug[ust] Busck”; 3 ♂♂: 1-“Bayamon | Jan[uary]1899”; 2- “Porto Rico | Aug[ust] Busck”; 1 ♀: 1-“Bayamon | Jan[uary]1899”, 2- “Porto Rico | Aug[ust] Busck”, 3-“GS-5620-SB | Argyria ♀ | lusella Z[eller] | det. Błeszyński, 19”, 4-“Genitalia slide | By SB ♀ | USNM 52861”; deposited in NMNH. 1 ♂ [abdomen in vial]: 1- “SYN-TYPE”, 2- “Bayamon | Jan 1899”, 3- “Porto Rico | Aug[ust] Busck”, 4- “Błeszyński | Collection | B.M. 1974-309”, 5- “Argyria | lusella Z[eller]. | ♂ | det. Błeszyński”, 6- “SYNTYPE | Argyria | diplomochalis | Dyar | det. M. Shaffer, 1975”, 7- “NHMUK013697137”; 1 ♂: 1- “SYN-TYPE”, 2- “Culebra I[sland]. | Feb 1899”, 3- “Porto Rico | Aug[ust] Busck”, 4- “Błeszyński | Collection | B.M. 1974-309”, 5- “SYN-TYPE” | Argyria | diplomochalis | Dyar | det. M. Shaffer, 1975”, 6- “NHMUK013697138”; deposited in the NHMUK.

##### Other specimens examined.

41 (see Suppl. material 2).

##### Morphological diagnosis.

Measuring 10–13 mm in wingspan this species (Figs 9, 14) is quite similar in size and forewing markings to *A.gonogramma* (Figs 8, 12), but it lacks a median line on the forewing and the median marginal markings are reduced to a faint brown bar on costa and a small dark-brown spot on the dorsal margin; the forewing costa and the head also appear more strongly marked (Fig. 31), notably more thickly dark brown at the base of the costa, on the frons laterally and on the labial and maxillary palpi; the costa of the forewing is also gold yellow to the apex, following the dark brown base. In male genitalia (Figs 19, 20), this species differs most noticeably from the others treated here in the thicker and less strongly bent apical section of the gnathos and in the short valva with a prominent sickle-shaped projection at its base. In female genitalia (Fig. 25), *A.diplomochalis* differs from the others treated here more noticeably by the long and narrow ductus bursae without sclerotised section as well as in the large, circular corpus bursae; the ostium region also lacks any sclerotised ‘pockets’.

**Figure 19.** Male genitalia of *Argyriadiplomochalis*. Holotype (NMNH).

##### Molecular results.

The phylogenetic clade corresponding to the species *A.diplomochalis* comprises only three samples sequenced for this study. It appears that no sequence available in the BOLD database corresponds to this species. All three species delimitation approaches identify this clade (Fig. 1) and the average genetic divergence with the two closest species, *A.insons* and *A.centrifugens*, are respectively 9.25% and 7.16%, which confirms the high divergence of this clade and its specific status. Within this species, the GMYC and PTP approaches separate CRA04 on one hand and CRA05 and CRA06 on the other; and a mean divergence of 2.87% is observed between the samples of this clade. But this divergence may be related to a geographical divergence since CRA04 comes from the island of Anguilla and CRA05-CRA06 come from the US Virgin Island of Saint Croix. The integration of a larger number of samples in a genetic study could allow a finer molecular characterisation of this species.

##### Distribution.

Antilles, from Cuba in the West to Dominica in the Lesser Antilles in the east (Fig. 35).

##### Remarks.

Described from 12 cotypes from Culebra Island and Bayamon, Puerto Rico, a lectotype is designated here to ensure that the name continues to refer to this species exclusively. Dyar (1913) stated “Cotypes, 12 specimens”, but only seven specimens were found at the NMNH while two others (now paralectotypes) are in the NHMUK.

Examination of specimens of “*A.diplomochalis*” cited by Kimball (1965), including ones in the FSCA labelled “5958,1” (Kimball’s number for that species), are *A.gonogramma* with scattered honey-brown scales on the forewings. This may be melanism caused by pupation during cold weather; all the specimens have been collected in winter or early spring.

#### ﻿ Argyria centrifugens

Taxon classificationAnimaliaLepidopteraCrambidae

Dyar, 1914

3F4CC2BB-6F2D-5E5F-A105-EA074702BCEE

**Figure 21.**
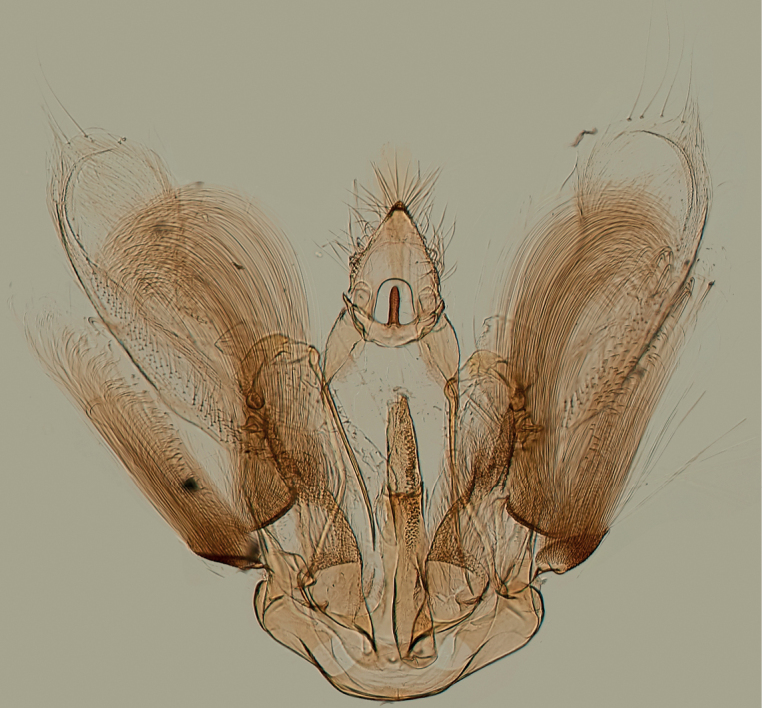
Male genitalia of *Argyriacentrifugens* (NMNH) holotype.

**Figure 22.**
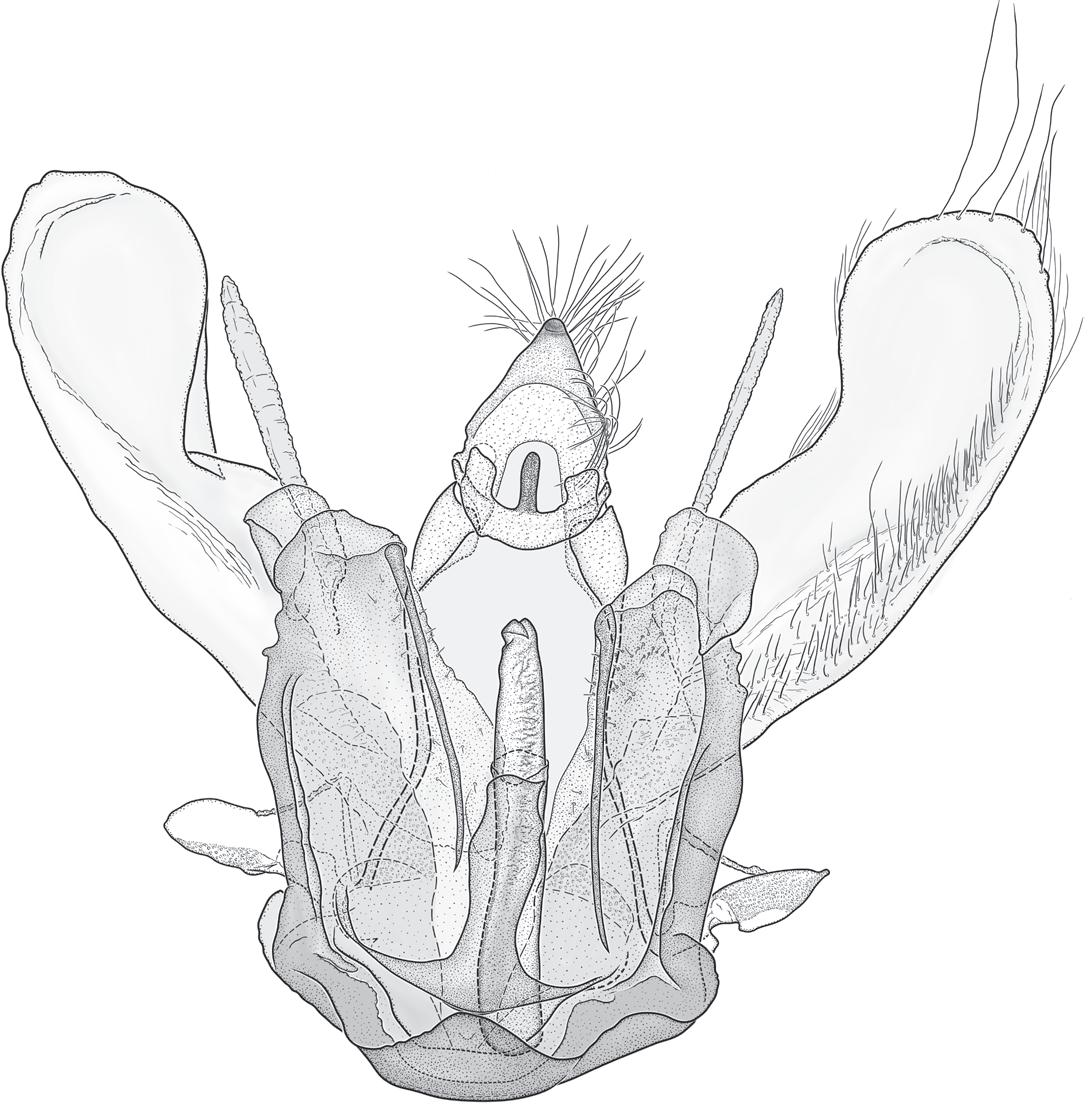
Male genitalia of *Argyriacentrifugens* holotype (NMNH); drawing without pheromone scales.

**Figure 23.**
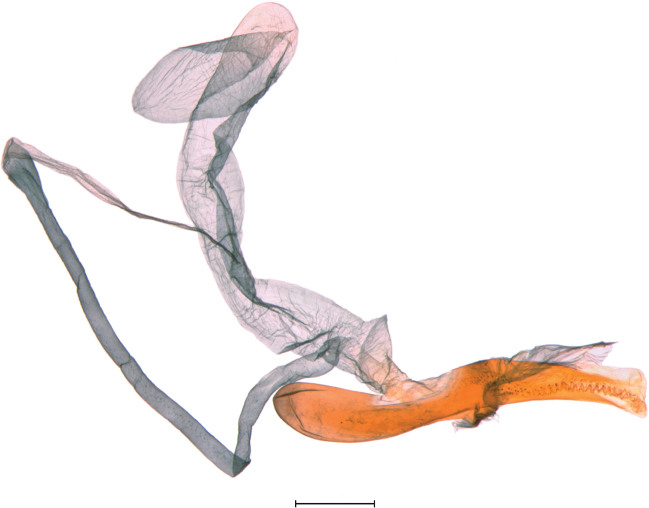
Male genitalia of *Argyriacentrifugens* (NMNH). Phallus in lateral view (Nicaragua, Selva Negra Ecolodge, MHNG-ENTO-13299). Scale bar: 250 µm.

Figs 10 , 21 , 22 , 23 , 26 , 32 , 35

Argyria centrifugens Dyar, 1914: 318. Type locality: Panama, Canal Zone, Paraiso. Błeszyński and Collins 1962: 212; Błeszyński 1967: 96; Munroe 1995: 35; Miller et al. 2012: 11; Landry et al. 2020: 101, fig. 6A.

##### Type material examined.

***Holotype*** ♂ (Figs 10, 21, 22), with label data as follows: 1- “ParaisoC[anal]Z[one] | Pan[ama]. Febr[uary].10.[19]11 | AugustBusck”, 2- “Type | No.16318 | U.S.N.M.”, 3- “Platytes | centrifugens| Type Dyar”, 4- “♂ genitalia | slide, 29Apr[il].[19]’32| C[arl].H[einrich]. #28”, 5- “Genitalia Slide | By 107,465 | USNM”; deposited in the NMNH.

##### Other specimens examined.

87 specimens (see Suppl. material 2).

##### Morphological diagnosis.

**Figures 24–26.**
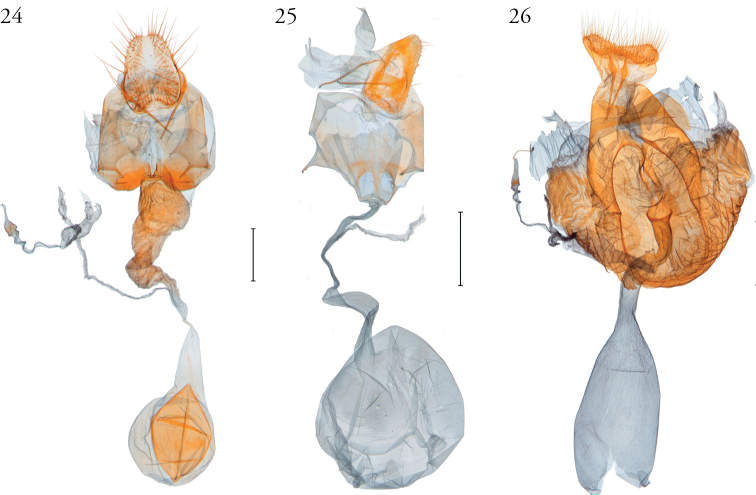
Female genitalia of *Argyria* species **24***Argyrialacteella* (holotype of *A.abronalis*, with spermatophore inside corpus bursae; OUMNH) **25***A.diplomochalis* (Anguilla Island, BL 1889, CMNH) **26***A.centrifugens* (Colombia, Amazonas, Leticia, MHNG-ENTO-97427). Scale bars: 250 µm (**24**), 500 µm (**25, 26**).

*Argyriacentrifugens* (Fig. 10) is very similar in wing markings to *A.lacteella* (Fig. 11), with which it can occur in sympatry in Central and South America, although the median line is always thin and not more pronounced in the middle or wide as in some South American specimens of *A.lacteella* (Fig. 7). It is also a bigger species, sporting a wingspan of 16 (male holotype) –17 mm in males and 16–19 mm in females, compared to 9.5–12.0 mm in males and 11.0–14.0 mm in females of *A.lacteella*. Apart from size these two species differ in the colouration of their labial palpi as those of *A.lacteella* (Fig. 29) and *A.gonogramma* (Fig. 30) are pale greyish brown and yellowish gold with the apex satiny white whereas those of *Argyriacentrifugens* (Fig. 32) are mostly dark brown with paler scales on the first palpomere but with the third palpomere dark brown to slightly paler brown. Both species are also very different in genitalia. The male genitalia of *A.centrifugens* (Figs 21–23) differ most notably in the three-pronged gnathos, the wider valva with a widely rounded apex and without a short hook-like projection at base but with a large membranous structure sporting a thin and pointed rod about half as long as the valva, directed toward the base of the valva and apparently articulated. The entire female genitalia are about twice as long in *A.centrifugens* (Fig. 26) than in *A.lacteella* (Fig. 24), the ostium is surrounded by a broad chamber with sclerotised wrinkles on the ventral wall, and the ductus bursae at the base is a medium-sized, thickly sclerotised tube in *A.centrifugens* whereas in *A.lacteella* the antrum consists of two lateral pockets of medium size and the base of the ductus bursae is a lightly sclerotised and corrugated round pocket.

##### Molecular results.

Phylogenetic inference reveals that the species *A.gonogramma* constitutes a distinct lineage separate from the species *A.insons*. The three species delimitation methods identified this species but also identified a subcluster separating the sample BLDNA141. This specimen originates from Colombia while all the other specimens come from Costa Rica. The observed genetic divergence is certainly related to a geographical divergence. A genetic study including samples from more distant localities such as Brazil would better characterise the genetic variability of this species.

##### Distribution.

Central and South America, from Honduras to Colombia and Brazil. Records from the central west coast of Florida are possibly recent introductions (Fig. 35).

##### Remarks.

The species was described from a specimen labelled “Type” of an unspecified sex and two other specimens, “Also two others, Cabima, May, 1911 (Busck).” (Dyar 1914). This “Type” is here considered the holotype. There is some variation observed in the male genitalia, especially noticeably in the length of the median process of the gnathos and in the length of the thin pointed rod at the base of the valva.

One female specimen identifiable as *A.centrifugens* was collected in Florida (Largo, Pinellas County), 1 Feb. 1995, by J.-G. Filiatrault, deposited in the FSCA (MGCL #1112910). It differs from typical specimens in that the labial palpi are mostly yellowish brown with a few dark brown scales on the first and second palpomeres. However, the maculation is otherwise typical, and the genitalia have the same rugose circumostial chamber as described above.

**Figures 27, 28.**
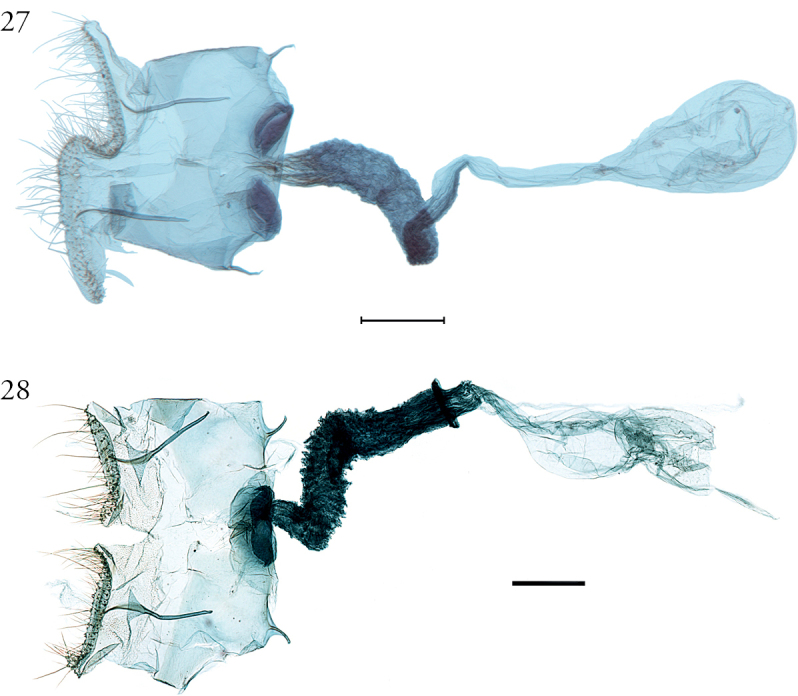
Female genitalia of *Argyria* species with ultimate segments cut dorsomedially from base to apex **27***Argyrialacteella* (USA, Florida, Glades Co.) **28***A.gonogramma* (USA, Florida, Orange Co.). Both in FSCA. Scale bar: 250 µm.

**Figures 29–32.**
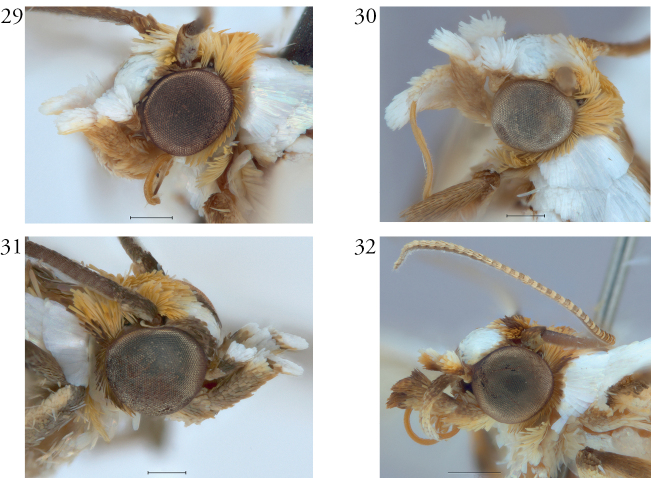
Heads of *Argyria* specimens **29***A.lacteella* (USA, Florida, Pinellas Co.) **30***A.gonogramma* (USA, Florida, Levy Co.) **31***A.diplomochalis* (US Virgin Islands, St Croix) **32***A.centrifugens* (Colombia, Amazonas, Leticia). All in MHNG. Scale bars: 250 µm (**29–31)**, 500 µm (**32**).

**Figure 33.**
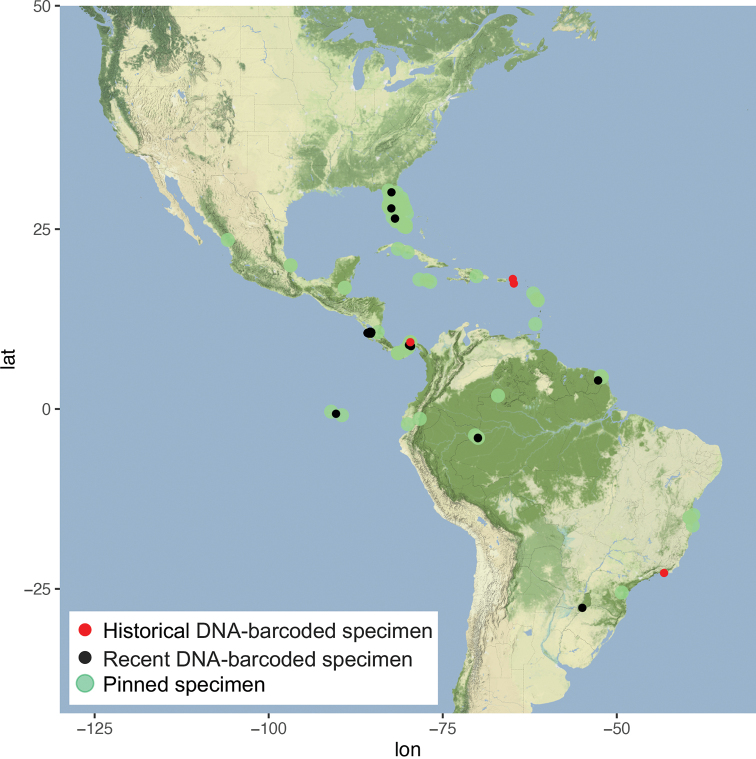
Distribution of *Argyrialacteella* (Fabricius).

**Figure 34.**
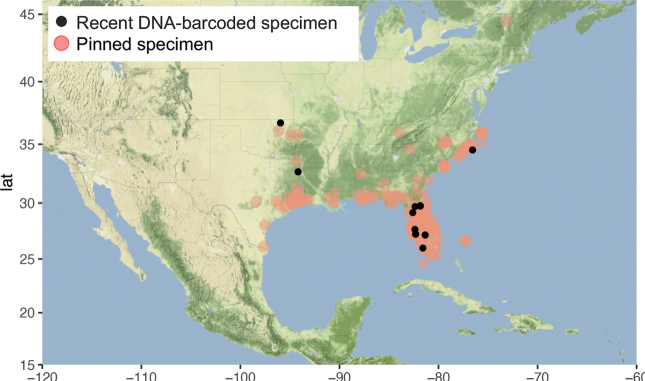
Distribution of *Argyriagonogramma* Dyar.

**Figure 35.**
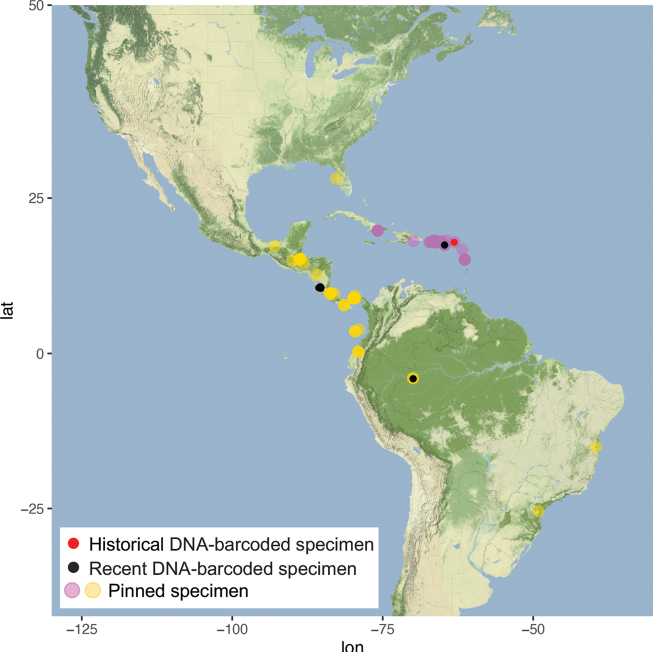
Distributions of *A.centrifugens* Dyar (yellow) and *A.diplomochalis* Dyar (pink).

## ﻿Discussion

We were able to resolve complex taxonomic questions for *Argyria* using an innovative DNA hybridisation capture protocol to recover high percentages of the DNA barcode of 18^th^–20^th^ century type specimens. Thus, we were able to solve taxonomic problems regarding synonymies of multiple names applied to the same species. Furthermore, we compiled distribution maps based on refined identities and specimens from multiple museums, leading to other questions regarding responses to environmental change through time. For example, we provided evidence to refine the type locality of *Argyrialacteella* as St Croix Island, whereas the three recent (2021) specimens we examined from that island belong to *A.diplomochalis* (see Suppl. material 2). The possible absence of *A.lacteella* on St Croix currently would not necessarily reflect the environmental situation of 235 years or so ago when the holotype was collected. Various reasons could explain why *A.diplomochalis* was recently collected on St Croix, instead of *A.lacteella*, including habitat change and/or destruction. A thorough moth collecting effort on the island may resolve the question of whether *A.lacteella* still occurs there. Much remains to be learned about this group of *Argyria* moths, especially about their biology and immature stages. Their distribution is also incompletely known and some specimen records, for example those of *A.gonogramma* in Vermont and of *A.centrifugens* in Florida, need further validation. Finally, many more taxonomic situations such as those dealt with in this paper occur in other insect groups that could be resolved using the innovative DNA hybridisation capture protocol presented here.

## Supplementary Material

XML Treatment for Argyria lacteella

XML Treatment for Argyria gonogramma

XML Treatment for Argyria diplomochalis

XML Treatment for Argyria centrifugens

## Supplementary materials

Supplementary material 1 Identity of *Argyrialacteella* (Fabricius) revised Data type word document https://binary.pensoft.net/file/806293This dataset is made available under the Open Database License (http://opendatacommons.org/licenses/odbl/1.0/). The Open Database License (ODbL) is a license agreement intended to allow users to freely share, modify, and use this Dataset while maintaining this same freedom for others, provided that the original source and author(s) are credited.Bernard Landry, Julia Bilat, James Hayden, M. Alma Solis, David C. Lees, Nadir Alvarez, Théo Léger, Jérémy Gauthier

Supplementary material 2 Species names, code numbers and collecting data Data type specimens names and collecting data Explanation note This table contains the species names, code numbers, and collecting data of all of the specimens examined in this study and that were used to generate the distribution maps. https://binary.pensoft.net/file/806294This dataset is made available under the Open Database License (http://opendatacommons.org/licenses/odbl/1.0/). The Open Database License (ODbL) is a license agreement intended to allow users to freely share, modify, and use this Dataset while maintaining this same freedom for others, provided that the original source and author(s) are credited.Bernard Landry, James Hayden, M. Alma Solis, David C. Lees, Théo Léger, Jérémy Gauthier
