# Supplementary material for: ﻿The identity of Argyrialacteella (Fabricius, 1794) (Lepidoptera, Pyraloidea, Crambinae), synonyms, and related species revealed by morphology and DNA capture in type specimens
Source: Zookeys. 2023 Feb 7;1146:1–42. doi: 10.3897/zookeys.1146.96099 (PMC10208364; doi:10.3897/zookeys.1146.96099)
Supplement: Supplementary material 1 — Identity of Argyrialacteella (Fabricius) revised [file zookeys-1146-001_article-96099__-s001.docx]

**Supplementary Material to** Identity of *Argyria lacteella* (Fabricius) revised

1. **DNA extractions of historical samples**
2. **Shotgun libraries preparation**
3. **Probe synthesis**
4. **Enrichment of shotgun libraries with hybridization/capture**
5. **DNA extractions of fresh samples with QIAamp DNA Micro Kit**
6. **PCR amplification of fresh DNA samples for Sanger sequencing**
7. **References**
8. **Acknowledgements**
9. **Appendices**

*Appendix 1: Information on extracted DNA*

*Appendix 2: Information on shotgun libraries*

*Appendix 3: Final POST-PCR product of shotgun library protocol*

*Appendix 4: Preparation of oligonucleotides solutions*

*Appendix 5: List of reagents*

# **DNA extractions of historical samples**

The protocol is based on Patzold, Zilli and Hundsdoerfer (2020).

**Non destructive lysis**

· Remove a leg with forceps and place it in a 1.5 mL tube. Sterilize the forceps on a Steri-250 (Keller; art.n°31100) before leg removal and between each specimen to prevent cross-contamination.

· Add 180 μL of Monarch gDNA Tissue Lysis Buffer and 20 μL of proteinase K. Mix the sample by gently inverting the tube several times and briefly centrifuge to collect droplets. Incubate overnight at 56°C in a Thermomixer at 300 rpm with a heated lid (e.g. ThermoMixerC, Eppendorf).

· Briefly centrifuge to collect droplets and gently pipet out the lysate and transfer it into a 1.5 mL tube for DNA purification.

*N.B.: The leg (abdomen, or other part of the specimen) can be rinsed with EtOH and dried after the lysis and minimal damage can be observed.*

**Destructive lysis**

· Remove a leg with forceps and place it in a 2 mL tube containing Stainless steel beads of 5 mm in diameter (Qiagen; art.n°69989). Sterilize the forceps on a Steri-250 before leg removal and between each specimen to prevent cross-contamination.

· Grind samples 2 times for 2 min at 24 Hz with a TissueLyser (Qiagen). Add 180 μL of Monarch gDNA Tissue Lysis Buffer and 20 μL of proteinase K. Mix the sample by gently inverting the tube several times and briefly centrifuge to collect droplets. Incubate overnight at 56°C in an incubator with a heated lid.

· Centrifuge the lysate at 16'000 x g for 1 min and gently pipet out the lysate and transfer it into a 1.5 mL tube for DNA purification.

**DNA purification**

· Add 2 volumes of DNA Cleanup Binding Buffer and mix well by pipetting up and down or flicking the tube, but do not vortex. Briefly centrifuge to collect droplets.

· Add 1.3 volumes of ≥96% Ethanol. Mix well by pipetting up and down and transfer 700 μL of the mix onto the column. Centrifuge for 3 min at 1'000 x g to bind the DNA to the membrane and discard the flow-through. Transfer the remaining mix into the column and centrifuge for 3 min at 1'000 x g. Perform a final centrifugation for 1 min at 16'000 x g and discard the flow-through.

· Add 500 μL of DNA Wash Buffer and spin for 1 min at 16'000 x g. Discard the flow-through.

· Place the column back into the collection tube and repeat this step.

· Place the Monarch DNA Cleanup column back into the collection tube and centrifuge for 2.5 min at 16'000 x g to dry the matrix.

· Transfer the Monarch DNA Cleanup column to a 1.5 ml low-bind tube. Pipet 13 μL of EB Buffer to the center of the matrix. Incubate for 10 min at room temperature. Centrifuge for 1 min at 16'000 x g.

· Pipet the eluate back onto the Monarch DNA Cleanup matrix and repeat the incubation and centrifugation steps.

Quantify the samples with the Qubit™ 1X dsDNA HS assay kit and assess the quality of the DNA with Fragment Analyzer (or Tapestation, or Bioanalyzer).

If necessary, dilute the samples to a maximum of 25-30 ng/μL and proceed with the shotgun libraries preparation protocol.

The concentration of purified DNA extracted from the leg(s) samples, either destructively or not, were estimated based on their fragment analyzer (FA) profiles or measured with a Qubit™ 1X dsDNA HS assay. As they were mainly ranging from ~0.1-3 ng/μL, no dilution was performed before proceeding to the shotgun library preparation. For CRA01 (DNA extracted with the non-destructive protocol from the abdomen), the concentration of DNA obtained was very high (53.6 ng/µL) and DNA was diluted to ~27 ng/μL before preparing the shotgun library. The DNA fragment sizes ranged from ~1 to ≤ 100 ng/μL, with a peak at ~40 bp. Sample CRA04, the most recently collected, showed a smear from 1 to 800 bp on FA, with a peak at ~100 bp. The DNA extracted from this leg also showed the highest yield (see Appendix 1 for details).

# **Shotgun libraries preparation**

The protocol is based on Suchan et al. (2016) with modifications detailed in the supplementary material of Toussaint et al. (2021). The reactions are briefly described here and the modifications compared to Toussaint et al*.* (2021) outlined in green. In summary, the modifications are the following: the first two purification steps were done with a Monarch DNA & PCR cleanup kit rather than with SPRI beads (ratio beads:sample of 2.7:1) to retain all the small DNA fragments ; for the same reason, the rest of the purification steps were performed with SPRI beads with higher ratios of beads:samples compared to the ratios used in Toussaint et al. (2021).

*N.B.: Unique dual-indexed (UDI) libraries were produced: different and unique P1-barcoded adapters and ILLPCR2 indexing primers were used for each sample (see Appendix 4 for details).*

*N.B*.: *Prepare all solutions (mixes) (and assemble all reactions) at room temperature if not stated otherwise. Samples should be stored at 4°C after each reaction, or at -20°C for long-term storage. Mix solutions (mixes) by pipetting up and down and flicking the tubes or shaking, but never vortex, unless stated otherwise.*

**1a) Phosphorylation**

Take 8 μL of DNA and add a mix of 1 μL of T4 polynucleotide kinase (10 U/μL) and 1 μL of T4 DNA Ligase Reaction Buffer (10X) for a total volume of 10 μL. Mix by flicking the tube and briefly centrifuge. Incubate for 30 min at 37°C, 20 min at 65°C.

**1b) Purification post-phosphorylation with Monarch DNA & PCR cleanup kit (5 μg)**

· Add 50 μL of EB buffer to the sample to reach a starting volume of 60 μL.

· Add 2 volumes of DNA Cleanup Binding Buffer (=120 μL) to each tube and transfer into a 1.5 mL tube.

· Mix well by pipetting up and down or flicking the tube. Do not vortex.

· Add 2 volumes of 100% Ethanol (=360 μL), mix by pipetting and transfer to the column.

· Centrifuge for 1 min at 16'000 x g. Discard the flow-through.

· Place the column into the collection tube. Add 200 μL of DNA Wash Buffer and centrifuge for 1 min at 16'000 x g. Discard the flow-through.

· Repeat the Wash Buffer step.

· Place the Monarch DNA Cleanup column back into the collection tube, and centrifuge for 2 min at 16'000 x g to dry the matrix.

· Transfer the Monarch DNA Cleanup column to a 1.5 mL and pipet 12 μL of EB Buffer into the center of the matrix. Incubate for 5 min at room temperature. Centrifuge for 1 min at 16'000 x g (or use gradual centrifugation with a final 1 min centrifugation step at 16'000 x g).

· Pipet the eluate back in the center of the matrix and repeat incubation and centrifugation steps.

· Transfer the eluted sample to a 0.2 mL tube.

**2) Heat denaturation**

Denaturate the sample at 95°C for 5 min then chill on ice to keep DNA in single strand conformation. Prepare a mix of water + ice blocks and place the 0.2 mL tube in it quickly after taking it out of 95°C.

**3) Guanidine tailing**

Prepare a mix with 4.7 μL of water, 2 μL of NEBuffer^TM^ 4 (10X), 2 μL of CoCl_2_ (2.5 mM), 0.8 μL of GTP (100 mM) and 0.5 μL of Terminal Transferase (TdT) (20 U/μL). Add the mix to the 10 μL of heat-denaturated sample for a total volume of 20 μL. Incubate for 30 min at 37°C and 10 min at 70°C.

**4) 2nd strand DNA synthesis**

Prepare a mix with 5.4 μL of water, 1 μL of NEBuffer^TM^ r 4 (10X), 0.6 μL of dNTPs (25 mM each), 1 μL of P2-CCCC oligonucleotide (15 μM) and 2 μL of Klenow Fragment (3'→5' exo-) (5 U/μL). Add the mix to the 20 μL of guanidine-tailed sample for a total volume of 30 μL. Incubate for 3 hours at 23°C and 20 min at 75°C.

**5a) Blunt-end reaction**

On ice, prepare a mix with 3.95 μL of water, 0.5 μL of NEBuffer^TM^ 4 (10X), 0.35 μL of BSA (10 mg/mL) and 0.2 μL of T4 DNA polymerase (3 U/μL). Still on ice, add the mix to the 30 μL of 2nd strand DNA synthesis sample for a total volume of 35 μL. Incubate for 15 min at 12°C. Store at 4°C. Only bring to room temperature shortly before the purification step.

**5b) Purification post-blunt-end reaction with Monarch DNA & PCR cleanup kit (5 μg):**

Follow the same protocol than for the purification post-phosphorylation (point 1b), except that only 25 μL of EB buffer is added in the first step to reach a starting volume of 60 μL.

**6a) Ligation of P1-barcoded adapters**

Prepare a mix with 6 μL of water, 2 μL of T4 DNA Ligase Buffer (10X) and 1 μL of T4 DNA Ligase (400 U/μL). Add the mix to the 10 μL of purified post-blunt-end reaction sample. Add 1 μL of P1-barcoded adapters solution (25 μM) for a total volume of 20 μL. Incubate overnight at 16°C.

**6b) Purification post-ligation reaction with SPRI beads**

Purify the sample with SPRI beads according to the producer's protocol with a beads:sample ratio of 1.4:1. Elute DNA in 20 μL of EB buffer.

**7a) Adapter fill-in**

Prepare a mix with 14.1 μL of water, 4 μL of ThermoPol reaction buffer (10X), 0.4 μL of dNTPs (25 mM each) and 1.5 μL *Bst* DNA polymerase, large fragment (8 U/μL). Add the mix to the 20 μL of purified post-ligation reaction sample for a total volume of 40 μL. Incubate for 20 min at 37°C.

**7b)** **Purification post-ligation reaction with SPRI beads**

Purify the sample with SPRI beads according to the producer's protocol with a beads:sample ratio of 2:1. Elute DNA in 20 μL of EB buffer.

**8a) Indexing PCR**

Prepare a mix with 14.1 μL of water, 10 μL of Phusion HF Buffer (5X), 0.4 μL of dNTPs (25 mM each) and 0.5 μL of Phusion U Hot Start DNA Polymerase (2 U/μL). Add 25 μL of the mix to the 20 μL of purified post-adapter fill-in sample. Add 5 μL of ILLPCR2 indexing primer solution (5 μM) for a total volume of 50 μL and mix well. Divide it into two 0.2 mL tubes of 25 μL each. Perform the following 2-steps PCR protocol on a thermocycler with heated lid at 99°C (*ramping = 4°C/s*): incubate at 98°C for 30 s; 30 cycles of 98°C for 7 s and 72°C for 25 s; final elongation for 7 min at 72°C. Pool the 2 PCR reactions together for a total volume of ~50 μL.

**8b)** **Purification of post-PCR reaction with SPRI beads**

Purify the sample with SPRI beads according to the producer's protocol with a beads:sample ratio of 1:1. Elute DNA in 20 μL of water.

POST-PCR DNA concentrations were measured with the Qubit™ 1X dsDNA HS assay kit and ranged from ~4-16 ng/μL (see Appendix 2 for details). A drawing of the structure of shotgun libraries can be found in Appendix 3.

# **Probe synthesis**

Final probe sets were ordered from Integrated DNA Technologies (IDT) using the "50 pmol o-Pool Oligos" option. Both sets consisted of 21 different 133 bases-long oligonucleotides: 108 bases of reference sequence + 25 bases of T7 promoter sequence. The two sets of biotinylated RNA probes synthetized are referred to as "forward probe set" and "reverse probe set".

Anneal each DNA oligonucleotide probe set to the T7 promoter reverse-complement oligonucleotide in a 20 μL reaction containing annealing buffer, 0.5 μM of each of the 21 oligonucleotide and 10.5 μM of the T7 promoter reverse-complement oligonucleotide (oligonucleotide were previously resuspended in EB buffer to make stock solutions). Heat each reaction at 95°C for 5 min and anneal into dsDNA by decreasing to 20°C with a down-ramping of 0.1°C/s on a thermocycler with heated lid.

Transcribe into biotinylated RNA probes following HiScribe T7 High Yield RNA Synthesis Kit protocol for "RNA synthesis with Modified Nucleotides" a total of 1 μg of annealed oligonucleotide for each probe set. Use Biotin-16-UTP (10 mM) as the Modified UTP and incubate the reaction for 2 hours at 37°C. Add 1 μL of TURBO™ DNase (2 U/μL) to the RNA probes and incubate 15 min at 37°C. Purify the RNA with RNeasy Mini Kit following a modified version of the "RNA cleanup" protocol: add 675 μL instead of 250 μL of 100% ethanol to the diluted RNA before transferring to the purification column to increase the recovery of small RNA fragments; elute the RNA in 20 μL of RNase-free water; add 1 μL of SUPERase-In™ RNase Inhibitor (20 U/μL) to the purified RNA probes sets.

The concentrations of both probes sets were measured with the Qubit™ RNA HS Assay Kit.

# **Enrichment of shotgun libraries with hybridization/capture**

The five shotgun libraries produced were quantified with the Qubit™ 1X dsDNA HS assay kit and pooled equimolarly based on their concentration.

*N.B*.: *Prepare all solutions (mixes) (and assemble all reactions) at room temperature if not stated otherwise. Samples should be stored at 4°C after each reaction, or at -20°C for long-term storage. Mix solutions (mixes) by pipetting up and down and flicking the tubes or shaking, but never vortex, unless stated otherwise.*

**Hybridization/capture with "forward probe set"**

Some of the equimolar pool was concentrated with a speedvac at 40°C on Heat+IR mode and centrifugation at 2500 rpm (IR vacuum centrifugal concentrator, catalog n°: NB-503CIR, N-BIOTEK) to a solution of ~14.3 ng/μL total concentration. Thus, 50 ng of the "forward probe set" were used to capture 100 ng of the concentrated libraries in two separate reactions at 55°C following the hybridization-capture protocol based on the Arbor Biosciences Custom protocol:

Prepare the hybridization mix by mixing 9 μL of SSPE (20X), 0.5 μL of EDTA (500 mM), 3.5 μL of Denhardt's solution (50X), 3.5 μL of of SUPERase-In™ RNase Inhibitor (20 U/μL), 0.4 μL of water and 5.1 μL of biotinylated RNA probes solution. Add 0.5 μL of SDS (10%) for a total volume of 22.5 μL and incubate the mix at 65°C in an incubator with heated lid for 5-10 min until dissolution of SDS. Leave the tube with the hybridization mix at room temperature.

Prepare the blocking mix by mixing 2.5 μL of Human Cot-1 DNA (1 mg/mL), 2.5 μL of Salmon sperm DNA (1 mg/mL) and 0.5 μL (= 1 μg total) of RNA blocking oligos BO.P5/BO.P7 for a total volume of 5.5 μL.

Add 5 μL of blocking mix to 7 μL of concentrated shotgun libraries (=~100 ng) and incubate for 5 min at 95°C in a thermocycler with heated lid at 99°C, then decrease the temperature of the block to 55°C. Place the hybridization mix on the thermocycler and incubate for 5 min at 55°C. Add 18 μL of the heated hybridization mix (=~50 ng of biotinylated RNA probes) to the 12 μL of blocking mix and shotgun libraries for a total volume of 30 μL. Mix and transfer to another thermocycler and incubate at 55°C, with heated lid at 65°C, for 40 hours.

Take 30 μL of Dynabeads™ M-280 Streptavidin, place on the magnetic rack and remove supernatant. Take out of the magnetic rack, wash with 200 μL of TEN buffer and mix by pipetting, place on the magnetic rack and remove the supernatant. Perform a total of three washes with 200 μL of TEN buffer and resuspend in 70 μL of TEN buffer. Incubate at 55°C until use.

*N.B.: The preparation of the Dynabeads™ M-280 Streptavidin should be done the same day that they are used.*

Prepare the wash buffer in excess to account for evaporation and pipetting errors during the preparation of aliquots. For 1 mL of wash buffer solution, mix 8 μL of SDS 10%, 200 μL of 0.1 X SSC/0.1% SDS solution and complete with water up to 1 mL. Aliquot the wash buffer in 0.2 mL tubes containing 185 μL of wash buffer and incubate at 55°C at least 45 minutes before use.

*N.B.: The preparation of the wash buffer and of the 0.1 X SSC/0.1% SDS solution should be done the same day that they are used.*

After 40 hours of hybridization, add 70 μL of pre-heated Dynabeads™ M-280 Streptavidin to capture the 30 μL of libraries and probes in a total volume of 100 μL. Vortex, short-spin and incubate for 30 min at 55°C. Vortex and centrifuge every 10 min. Place on the magnetic rack, remove the supernatant, add 180 μL of pre-heated wash buffer, vortex, short-spin, then incubate for 10 min at 55°C. Repeat this washing step four times then resuspend in 30 μL of EB buffer and store at RT before the post-capture amplification.

Prepare the post-capture amplification master mix by mixing 10 μL of IS5/IS6 primer solution (5 μM each oligonucleotide) with 10 μL of water and 50 μL of KAPA HiFi HotStart ReadyMix. Add 70 μL of post-capture amplification master mix to 30 μL of captured libraries (still containing the Dynabeads™ M-280 Streptavidin) for a total volume of 100 μL. Mix and divide in two PCR replicates of 50 μL each. Perform the following 3-steps PCR protocol on a thermocycler with heated lid at 99°C (*ramping = 4°C/s*): incubate at 98°C for 2 min; 15 cycles of 98°C for 20 s, 65°C for 30 s and 72°C for 30 s; final elongation for 5 min at 72°C. Pool the two PCR replicates and purify with SPRI beads according to the producer's protocol with a beads:sample ratio of 0.8:1. Elute DNA in 15 μL of EB buffer.

The two separate hybridization/capture reactions with the "forward probe set" were pooled and concentrated with SPRI beads to 9 μL (in EB buffer) according to the producer's protocol with a beads:sample ratio of 0.8:1. The concentration measured with the Qubit™ 1X dsDNA HS assay kit was ~0.9 ng/μL.

7 μL (= 6.1 ng) of the captured-enriched libraries pool was used to perform a second round of hybridization/capture enrichment at 65°C with 50 ng of the "forward probe set". The protocol was the same than with the hybridization/capture enrichment at 55°C, except for the hybridization, capture with Dynabeads™ M-280 Streptavidin, and wash steps, which were performed at 65°C instead of 55°C. Twenty PCR cycles instead of 15 were used for the post-capture amplification. Libraries were eluted in 15 μL of EB buffer after purification with SPRI beads. The concentration measured with the Qubit™ 1X dsDNA HS assay kit was ~90 ng/μL.

**Hybridization/capture with "reverse probe set"**

The remaining volume of equimolar pool of shotgun libraries was concentrated with SPRI beads according to the producer's protocol with a beads:sample ratio of 0.8:1 and eluted in 9 μL of EB buffer to maximize the total ng input of shotgun libraries for the hybridization reaction.

The protocol for hybridization/capture was the same than the one used for the hybridization/capture with the "forward probe set" except for the following details:

only one reaction was performed for the hybridization/capture at 55°C and due to the limited material available, only 24.5 ng of concentrated pool of shotgun libraries was used with 50 ng of biotinylated RNA probes.

The concentration of the 15 μL of captured libraries after hybridization/capture at 55°C and enrichment with 15 PCR cycles was ~0.7 ng/μL. Thus, 4.9 ng (7 μL) of the captured-enriched libraries pool was used to perform a second round of hybridization/capture enrichment at 65°C with 50 ng of the "reverse probe set". Only 15 PCR cycles instead of 20 were used to amplify the libraries after the hybridization/capture at 65°C. The concentration measured with the Qubit™ 1X dsDNA HS assay kit was ~13 ng/μL.

# **DNA extractions of fresh samples with QIAamp DNA Micro Kit**

DNA from two specimens (CRA05 and CRA06) collected in 2021 were extracted with a QIAamp DNA Micro Kit following the protocol for isolation of genomic DNA from tissues with some minor modifications:

· Remove and grind the leg with the same protocol as described above for historical specimens prior to the addition of 180 μL of Buffer ATL and 20 μL of proteinase K.

· After overnight incubation, centrifuge the tube and transfer the lysate to a new 1.5 mL tube taking care not to pipet any non-digested tissues fragments.

· After addition of ethanol, mix the sample by pipetting up and down and transfer directly to the column.

· Elute the DNA in 25 μL of EB buffer and incubate 5-10 min instead of 1 min.

The concentration measured with a Qubit™ 1X dsDNA HS assay kit was ~0.8 ng/μL for both samples. The profiles obtained with a High Sensitivity Fragment Analyzer 1-6000 bp kit showed a good quality of the DNA with fragments ≥ 6 kb and no fragments < 700 bp.

For the samples BLDNA065, BLDNA137, BLDNA138 and BLDNA141, DNA was extracted using the Macherey-Nagel DNA extraction kit (Dürren, Germany) at the MFNB.

# **PCR amplification of fresh DNA samples for Sanger sequencing**

The COI barcode was amplified by PCR with the primers pair used in the study of Landry and Andriollo (2020) referred to as H02198 and COImod. PCR amplification parameters needed to be adjusted in regard to the use of so-called "universal primers" and also to the amplification of small amount of DNA. The two following protocols proved to be successful and the required adjustments of PCR parameters are outlined below. PCR samples were purified with SPRI beads according to the producer's protocol with a beads:sample ratio of 0.8:1; DNA was eluted in 10 μL of EB buffer.

**Final PCR protocol with MyFi™ Mix (used for CRA05)**

Assemble a 20 μL PCR reaction containing 10 μL of MyFi™ Mix (2X), 0.6 μL of each primer solution (20 μM), ~1 ng of DNA and water. Perform the following PCR in a thermocycler with heated lid at 99°C and ramping rate at 2°C/s: 3 min of initial denaturation at 95°C, 40 cycles of 30 s denaturation at 94°C, 45 s of annealing at 50°C, 60 s of elongation at 72°C and final elongation for 10 min at 72°C.

Adjustments of PCR parameters compared to a classic PCR with MyFi™ Mix and to the protocol from Landry and Andriollo (2020):

· Ramping rate reduced to 2°C/s for all steps of the PCR.

· Increase of primer concentration to 0.6 μM each instead of 0.4 μM each.

· Increase of initial denaturation time to 3 min at 95°C.

· Increase total number of cycles to 40.

· Decrease of denaturation temperature from 95°C to 94°C in PCR cycles.

· Reduction of annealing temperature to 50°C.

· In PCR cycles: denaturation time set to 30 s, annealing time extended to 45 s, extension time extended to 60 s.

· Final extension time extended to 10 min.

**Final PCR protocol with Platinum™ SuperFi™ DNA Polymerase (used for CRA06)**

Assemble a 10 μL PCR reaction containing 2 μL of SuperFi™ Buffer (5X), 0.8 μL of dNTPs (2.5 mM each), 0.5 μL of each primer solution (20 μM), 2 μL 5X SuperFi™ GC Enhancer, 0.1 μL of Platinum™SuperFi™ DNA Polymerase (2 U/μL), ~1 ng of DNA and water. Perform the following PCR in a thermocycler with heated lid at 99°C and ramping rate at 2°C/s: 1 min of initial denaturation at 98°C, 40 cycles of 10 s denaturation at 95°C, 30 s of annealing at 50°C, 30 s of elongation at 72°C and final elongation for 10 min at 72°C.

Adjustments of PCR parameters compared to a classic PCR Platinum™ SuperFi™ DNA Polymerase

· Ramping rate reduced to 2°C/s for all steps of the PCR.

· Increase of primers concentration to 1 μM each instead of 0.5 μM each.

· Increase of initial denaturation time to 1 min at 98°C.

· Increase total number of cycles to 40.

· Decrease of denaturation temperature from 98°C to 95°C in PCR cycles.

· Annealing temperature reduced to 50°C.

· In PCR cycles: denaturation time set to 10 s, annealing time extended to 30 s, extension time extended to 30 s.

· Final extension time extended to 10 min.

For the samples BLDNA065, BLDNA137, BLDNA138 and BLDNA141, the COI barcode region was amplified using the LCO/Nancy primer combination. For samples where amplification failed, two fragments COI-1a (LCO/K699) and COI-1b (COIf220/Nancy) were amplified subsequently. The PCR-mix is composed of 17.80µl of H20, 0.50µl of forward primer (10µM), 0.50µl of reverse primer (10µM), 0.50µl of dNTPs, 1.00µl of Mg, 2.50µl of buffer and 0.20µl of Taq Polymerase. PCR program is composed of an initial temperature of 95°C during 5min followed by 42 cycles of 30s at 95°C, 40s at 49°C, 50s at 72°C and a final step of 10min at 72°C. Amplification results were checked on a 1% agarose gel. Sequencing was done by Macrogen (The Netherlands) in both directions. Sequences were eye-checked and aligned using Phyde 0.9971 (Müller et al., 2005).

# **References**

Landry B, Andriollo T (2020) A review of the genus *Microcrambus* Błeszyński, 1963 (Lepidoptera, Pyraloidea, Crambinae) in Colombia, with descriptions of two new species. Revista UDCA Actualidad & Divulgación Científica 23(2): e1628.<https://doi.org/10.31910/rudca.v23.n2.2020.1628>

Müller J, Müller K, Neinhuis C, Quandt D (2005) PhyDE-Phylogenetic Data Editor.

Patzold F, Zilli A, Hundsdoerfer AK (2020) Advantages of an easy-to-use DNA extraction method for minimal-destructive analysis of collection specimens. PloS one 15(7): e0235222.<https://doi.org/10.1371/journal.pone.0235222>

Suchan T, Pitteloud C, Gerasimova NS, Kostikova A, Schmid S, Arrigo N, Pajkovic M, Ronikier M, Alvarez N (2016) Hybridization capture using RAD probes (hyRAD), a new tool for performing genomic analyses on collection specimens. PloS one 11(3): e0151651.<https://doi.org/10.1371/journal.pone.0151651>

Toussaint EF, Gauthier J, Bilat J, Gillett CP, Gough HM, Lundkvist H, Blanc M, Muñoz-Ramírez CP, Alvarez N (2021) HyRAD-X exome capture museomics unravels giant ground beetle evolution. Genome biology and evolution 13(7): evab112.<https://doi.org/10.1093/gbe/evab112>

# **Acknowledgements**

We thank Marjorie Labédan (University of Lausanne) for performing the tests to adapt the shotgun library protocol to our very degraded DNA samples extracted with the Monarch DNA & PCR cleanup kit, i.e. purification tests with either SPRI-beads or Monarch DNA & PCR cleanup kit in steps 1b, 5b and 6b of the shotgun library protocol. We thank Hélène Mottaz (MHNG) for her help in revising this protocol.

# **Appendices**

## **Appendix 1: Information on extracted DNA**

| **ID** | **Tissue** | **Year of collecting** | **Extraction method** | **DNA conc. in ng/μL (based on)** | **DNA fragment sizes: range in bp** | **DNA fragment sizes: peak in bp** |
| --- | --- | --- | --- | --- | --- | --- |
| CRA01 | abdomen | before 1859 | non-destructive | 53.6 (Qubit) | 1 to 100 | 30-35 |
| CRA02 | leg | before 1863 | non-destructive | 0.4 (FA) | 1 to 100 | 30-35 |
| CRA03 | leg | 1784-1789 | destructive | 0.5 (FA) | 1 to 100 | 30-35 |
| CRA04 | leg | 1992 | destructive | 2.94 (FA) | 1 to 800 | 100 |
| CRA07 | 2 legs | 1911 | destructive | 0.17 (FA) | 1 to 100 | 30-35 |

## **Appendix 2: Information on shotgun libraries**

| **ID** | **DNA conc. in ng/μL POST-PCR measured with Qubit** |
| --- | --- |
| CRA01 | 15.8 |
| CRA02 | 6.67 |
| CRA03 | 5.91 |
| CRA04 | 4.67 |
| CRA07 | 4.72 |

**Appendix 3: Final POST-PCR product of shotgun library protocol**


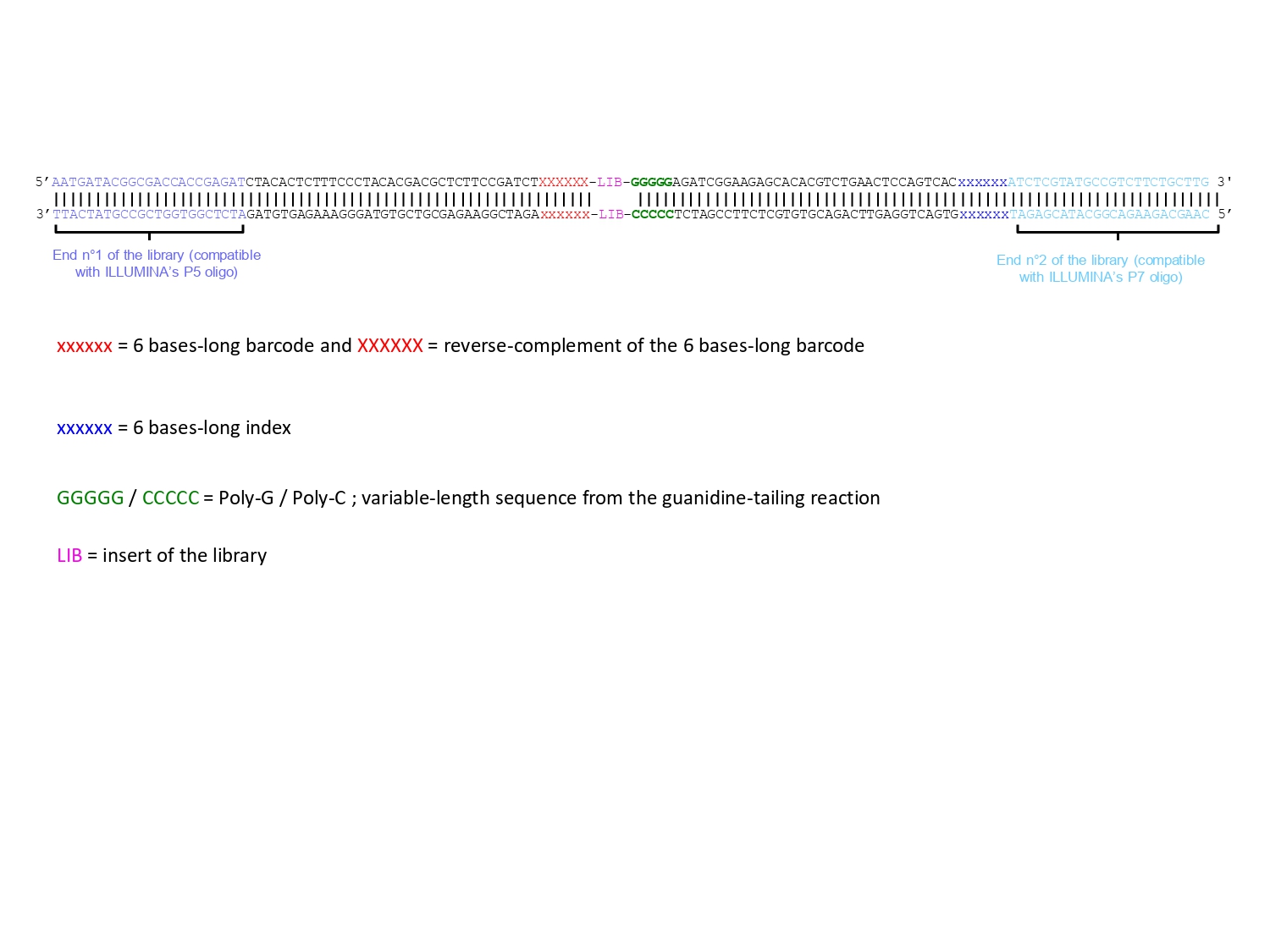


## **Appendix 4: Preparation of oligonucleotides solutions**

P2-CCCC solution (15 μM):

**Sequence 5'->3' P2-CCCC oligonucleotide:** GTGACTGGAGTTCAGACGTGTGCTCTTCCGATCTCCCCC

**Preparation:**

Prepare a 15 μM solution in Tris-HCl 10 mM.

P1-barcoded adapters solution (25 μM)

**Sequence 5'->3' P1-X.1-barcoded oligonucleotide:** ACACTCTTTCCCTACACGACGCTCTTCCGATCTXXXXXX

**Sequence 5'->3' P1-X.2-barcoded oligonucleotide:**

xxxxxxAGATCGGAAGAGC

where xxxxxx = 6 bases-long barcode and XXXXXX = reverse-complement of the 6 bases-long barcode.

**Preparation**

Prepare a 25 μM each oligonucleotide solution in annealing buffer. Heat at 95°C for 5 min and anneal into dsDNA by decreasing to 20°C with a down-ramping of 0.1°C/s on a thermocycler with heated lid. Prepare a different solution using a different barcode sequence for each sample if libraries are designed in UDI.

ILLPCR2 indexing primer solution (5 μM)

**Sequence 5'->3' ILLPCR1 oligonucleotide:**

AATGATACGGCGACCACCGAGATCTACACTCTTTCCCTACACGACG

**Sequence 5'->3' ILLPCR2_X oligonucleotide:**

CAAGCAGAAGACGGCATACGAGATxxxxxxGTGACTGGAGTTCAGACGTGTGC

where xxxxxx = 6 bases-long index

**Preparation**

Prepare a 5 μM each oligonucleotide solution in Tris-HCl 10 mM.

RNA blocking oligos BO.P5/BO.P7 (2 μg total/μL)

**Sequence 5'->3' T7 promoter oligonucleotide:**

AGTACTAATACGACTCACTATAGG

**Sequence 5'->3' BO.P5 oligonucleotide:**

AGATCGGAAGAGCGTCGTGTAGGGAAAGAGTGTAGATCTCGGTGGTCGCCGTATCATTCCCTATAGTGAGTCGTATTAGTACT

**Sequence 5'->3' BO.P7 oligonucleotide:**

AGATCGGAAGAGCACACGTCTGAACTCCAGTCACNNNNNNATCTCGTATGCCGTCTTCTGCTTGCCCTATAGTGAGTCGTATTAGTACT

where NNNNNN = any base (IUPAC nucleotide code)

**Preparation**

Prepare a solution of T7 promoter and BO.P5 10 μM each oligonucleotide in annealing buffer. Heat at 95°C for 5 min and anneal into dsDNA by decreasing to 20°C with a down-ramping of 0.1°C/s on a thermocycler with heated lid. Transcribe 0.7 μL of annealed oligonucleotide solution into RNA following HiScribe T7 High Yield RNA Synthesis Kit protocol for "RNA synthesis with Modified Nucleotides" with a 2 hours incubation at 37°C. Add 1 μL of TURBO™ DNase (2 U/μL) to the RNA and incubate 15 min at 37°C. Purify the RNA with RNeasy Mini Kit following a modified version of the "RNA cleanup" protocol: add 675 μL instead of 250 μL of 100% ethanol to the diluted RNA before transferring to the purification column to increase the recovery of small RNA fragments; elute the RNA in 20 μL of RNase-free water; add 1 μL of SUPERase-In™ RNase Inhibitor (20 U/μL) to the purified BO.P5 RNA blocking solution.

Follow the same protocol with T7 promoter and BO.P7 oligonucleotide to obtain a BO.P7 RNA blocking solution.

Quantify both BO.P5 and BO.P7 RNA blocking solutions with a Qubit^TM^ RNA HS assay kit and prepare an equimolar pool of 2 μg total RNA per μL to be used as RNA blocking oligos BO.P5/BO.P7 solution in the hybridization/capture protocol.

IS5/IS6 primer solution (5 μM)

**Sequence 5'->3' IS5 oligonucleotide:**

AATGATACGGCGACCACCGAGAT

**Sequence 5'->3' IS6 oligonucleotide:**

CAAGCAGAAGACGGCATACGAGAT

**Preparation:**

Prepare a 5 μM each oligonucleotide solution in Tris-HCl 10 mM.

Sanger sequencing primers (20 μM)

**Sequence 5'->3' H02198 oligonucleotide:**

TAAACTTCAGGGTGACCAAAAAATCA

**Sequence 5'->3' COImod oligonucleotide:**

AGTTCTAATCATAARGATATYGG

**Preparation**

Prepare a 20 μM solution in Tris-HCl 10 mM for both Sanger sequencing primers.

## **Appendix 5: List of reagents:**

| **REAGENT** | **CATALOG NUMBER** | **PRODUCER** |
| --- | --- | --- |
| Annealing buffer (100 mM Tris-HCl, 500 mM NaCl) | Homemade from stock solutions (Invitrogen) |  |
| Bio-16-UTP (10 mM ) | AM8452 | Invitrogen (Thermo Fisher Scientific) |
| BSA (10 mg/mL) | B9000S=20 mg/ml diluted to 10 mg/ml | New England Biolabs |
| *Bst* DNA Polymerase, Large Fragment (8 U/μL) | M0275S, M0275L | New England Biolabs |
| CleanNGS purification beads | CNGS0500 = 1 bottle 500 mL | CleanNA |
| CoCl2 (2.5 mM) | B0252S (provided with TdT M0315) | New England Biolabs |
| Denhardt’s solution 50X | 750018 | Invitrogen (Thermo Fisher Scientific) |
| dNTP Set, 100 mM Solutions | R0181, R0182, R0186 | Thermo Scientific |
| Dynabeads™ M-280 Streptavidin | 11205D, 11206D | Invitrogen (Thermo Fisher Scientific) |
| EB Buffer (=10 mM Tris-Cl, pH 8.5) | 19086 | QIAGEN |
| EDTA (0.5 M), pH=8 | R1021 | Thermo Scientific |
| EDTA (0.5 M), pH=8, Ultrapure | 15575020 | Invitrogen (Thermo Fisher Scientific) |
| Ethanol absolute anhydrous RPE - For analysis - ACS - Reag. Ph.Eur. - Reag. USP 2.5 L | 4146082 | Carlo Erba Reagents |
| GTP solution, Tris buffered (100 mM) | R1461 | Thermo Scientific |
| High Sensitivity NGS Fragment Analysis Kit (1 - 6000bp) | DNF-474-0500 | Agilent Technologies |
| HiScribe T7 High Yield RNA Synthesis Kit, 50 reactions | E2040S | New England Biolabs |
| Human Cot-1 DNA™ (1 mg/mL) | 15279011 | Invitrogen (Thermo Fisher Scientific) |
| KAPA HiFi HotStart Ready Mix,1.25 ml | KK2601 (07958927001), KK2602 (07958935001) | Kapa Biosystems (Roche Diagnostics) |
| Klenow Fragment (3'→5' exo-) (5 U/μL) | M0212S, M0212L | New England Biolabs |
| Molecular biology grade water HyClone 1 L | SH30538.03 | Cytiva Europe GmbH |
| Monarch DNA & PCR cleanup kit (5 μg) | T1030 | New England Biolabs |
| Monarch gDNA Tissue Lysis Buffer | T3010 | New England Biolabs |
| MyFi™ Mix | BIO-25049, BIO-25050 | Bioline |
| NaCl (5 M), RNase-free | AM9760G, AM9759 | Invitrogen (Thermo Fisher Scientific) |
| NEBuffer^TM^ 4 10X | B7004S | New England Biolabs |
| Phusion HF Buffer 5X | F-518 (provided with Phusion U Polymerase F555) | Thermo Scientific |
| Phusion U Hot Start DNA Polymerase (2 U/μL) | F555S, F555L | Thermo Scientific |
| Platinum™ SuperFi™ DNA Polymerase (2U/uL) | 12351010, 12351050, 12351250 | Invitrogen (Thermo Fisher Scientific) |
| Proteinase K (20 mg/mL) | 19131, 19133 | QIAGEN |
| QIAamp DNA Micro Kit (50) | 56304 | QIAGEN |
| Qubit™ 1X dsDNA HS assay Kit | Q33230, Q33231 | Invitrogen (Thermo Fisher Scientific) |
| Qubit™ RNA HS Assay Kit | Q32852, Q32855 | Invitrogen (Thermo Fisher Scientific) |
| RNeasy Mini kit | 74104, 74106 | Qiagen |
| Salmon Sperm DNA (1 mg/mL) | 15632011 | Invitrogen (Thermo Fisher Scientific) |
| SDS (10%), BioUltra, for molecular biology | 71736 | Sigma-Aldrich |
| SPRI beads -> CleanNGS purification beads | CNGS0500 = 1 bottle 500 mL | CleanNA |
| SSC 20X | 15557044, 15557036 | Invitrogen (Thermo Fisher Scientific) |
| SSPE 20X, Ultrapure | 15591043 | Invitrogen (Thermo Fisher Scientific) |
| SUPERase-In™ RNase Inhibitor (20 U/μL) | AM2694, AM2696 | Invitrogen (Thermo Fisher Scientific) |
| SuperFi™ Buffer 5X | 12355005, Provided with Platinum SuperFi DNA Polymerase | Thermo Scientific (Thermo Fisher Scientific) |
| SuperFi™ GC Enhancer 5X | Provided with Platinum SuperFi™ DNA Polymerase | Thermo Scientific (Thermo Fisher Scientific) |
| T4 DNA Ligase (400 U/μL) | M0202S, M0202L | New England Biolabs |
| T4 DNA Ligase Reaction Buffer 10X (10mM ATP) | B0202S (provided with T4 DNA Ligase M0202) | New England Biolabs |
| T4 DNA Polymerase (3 U/μL) | M0203S, M0203L | New England Biolabs |
| T4 Polynucleotide Kinase (10 U/ μL) | M0201S, M0201L | New England Biolabs |
| TEN (10 mM Tris-HCl, 1 mM EDTA, 1 M NaCl) | Homemade from stock solutions (Invitrogen) |  |
| Terminal Transferase (TdT) (20 U/ μL) | M0315S, M0315L | New England Biolabs |
| ThermoPol Reaction Buffer Pack 10X | B9004 (provided with *Bst* DNA Polymerase Large Fragment M0275) | New England Biolabs |
| Tris (1 M), pH 8.0, RNase-free | AM9855G, AM9856 | Invitrogen (Thermo Fisher Scientific) |
| TURBO™ DNase (2 U/μL) | AM2238, AM2239 | Invitrogen (Thermo Fisher Scientific) |
